# Long-Term Boron-Excess-Induced Alterations of Gene Profiles in Roots of Two Citrus Species Differing in Boron-Tolerance Revealed by cDNA-AFLP

**DOI:** 10.3389/fpls.2016.00898

**Published:** 2016-06-24

**Authors:** Peng Guo, Yi-Ping Qi, Lin-Tong Yang, Xin Ye, Jing-Hao Huang, Li-Song Chen

**Affiliations:** ^1^Institute of Plant Nutritional Physiology and Molecular Biology, College of Resources and Environment, Fujian Agriculture and Forestry UniversityFuzhou, China; ^2^Fujian Provincial Key Laboratory of Soil Environmental Health and Regulation, College of Resources and Environment, Fujian Agriculture and Forestry UniversityFuzhou, China; ^3^Institute of Materia Medica, Fujian Academy of Medical SciencesFuzhou, China; ^4^Pomological Institute, Fujian Academy of Agricultural SciencesFuzhou, China; ^5^The Higher Educational Key Laboratory of Fujian Province for Soil Ecosystem Health and Regulation, Fujian Agriculture and Forestry UniversityFuzhou, China

**Keywords:** boron-excess, cDNA-AFLP, *Citrus grandis*, *Citrus sinensis*, roots

## Abstract

Boron (B) toxicity is observed in some citrus orchards in China. However, limited data are available on the molecular mechanisms of citrus B-toxicity and B-tolerance. Using cDNA-AFLP, we identified 20 up- and 52 down-regulated genes, and 44 up- and 66 down-regulated genes from excess B-treated *Citrus sinensis* and *Citrus grandis* roots, respectively, thereby demonstrating that gene expression profiles were more affected in the latter. In addition, phosphorus and total soluble protein concentrations were lowered only in excess B-treated *C. grandis* roots. Apparently, *C. sinensis* had higher B-tolerance than *C. grandis*. Our results suggested that the following several aspects were responsible for the difference in the B-tolerance between the two citrus species including: (a) B-excess induced *Root Hair Defective 3* expression in *C. sinensis* roots, and repressed *villin4* expression in *C. grandis* roots; accordingly, root growth was less inhibited by B-excess in the former; (b) antioxidant systems were impaired in excess B-treated *C. grandis* roots, hence accelerating root senescence; (c) genes related to Ca^2+^ signals were inhibited (induced) by B-excess in *C. grandis* (*C. sinensis*) roots. B-excess-responsive genes related to energy (i.e., *alternative oxidase* and *cytochrome P450*), lipid (i.e., *Glycerol-3-phosphate acyltransferase 9* and *citrus dioxygenase*), and nucleic acid (i.e., *HDA19, histone 4*, and *ribonucleotide reductase RNR1 like protein*) metabolisms also possibly accounted for the difference in the B-tolerance between the two citrus species. These data increased our understanding of the mechanisms on citrus B-toxicity and B-tolerance at transcriptional level.

## Introduction

Boron (B), an essential element for normal growth and development of higher plants, is taken up by roots in the form of boric acid from soil solution. Despite this, B is toxic to higher plants when present in excess (Nable et al., 1997; Chen et al., 2012; Sang et al., 2015). Although B-toxicity is less common than B-deficiency, it is still a serious problem in many agricultural regions across the world. However, the molecular mechanisms on B-toxicity and B-tolerance in higher plants are not fully understood.

In higher plants, B-excess cause impairments of cellular functions and physiological and biochemical processes, such as plant growth (Ayvaz et al., 2012; Guo et al., 2014), hormone balance (Ayvaz et al., 2012, 2015), uptake and use efficiency of other mineral elements (Papadakis et al., 2003; Aquea et al., 2012), leaf photosynthetic electron transport chain (Han et al., 2009), photosynthetic rate (Han et al., 2009; Sheng et al., 2010; Chen et al., 2012; Guo et al., 2014), photosynthetic enzyme activities (Han et al., 2009), reactive oxygen metabolism (Keles et al., 2004; Cervilla et al., 2007; Han et al., 2009), chlorophyll (Chl) and carotenoid (Car) levels (Han et al., 2009; Ayvaz et al., 2012), cell wall biosynthesis (Huang et al., 2016); carbohydrate (Keles et al., 2004; Papadakis et al., 2004a,b; Han et al., 2009), energy (Reid et al., 2004; Guo et al., 2014), nucleic acid (Wang et al., 1995), and nitrogen (N; Cervilla et al., 2009; Chen et al., 2014) metabolisms, and leaf and root structure (Papadakis et al., 2004a,b; Chen et al., 2012; Huang et al., 2014; Mesquita et al., 2016).

Transcriptomic analysis not only offers us the chance to elucidate the molecular mechanisms on plant B-toxicity and B-tolerance but also is very useful for us to obtain B-excess-responsive genes possibly responsible for B-tolerance. There are several studies investigating B-excess-induced alterations of gene profiles in higher plants. Using microarray, Kasajima and Fujiwara (2007) isolated many high-B-induced genes from *Arabidopsis thaliana* roots and rosette leaves; nine of which were validated in an independent experiment. Using suppression subtractive hybridization (SSH), Hassan et al. (2010) found 111 up-regulated clones showing similar to known proteins from high-B-treated roots of the B-tolerant barely bulk, thereby suggesting that both polyamines and ascorbate–glutathione pathway played a role in the B-tolerance of barely. Padmanabhan et al. (2012) used SSH to isolate 219 and 113 unique B-excess-responsive non-redundant expressed sequence tags (ESTs) from extremely B-tolerant *Puccinellia distans* and moderately B-tolerant *Gypsophila arrostil* plants, respectively. In addition to restricting B accumulation in plant tissues *via* enhancing the expression of *efflux transporters* similar to barely *Bot1*, genes related to stress/defense, protein synthesis, cellular organization, and signal transduction had a positive role in the B-tolerance of *P. distans*. Aquea et al. (2012) obtained 240 up-regulated genes, which mainly function in ABA signaling, ABA response, or cell wall modifications, and 211 down-regulated genes, which are mainly related to glucosinolate biosynthesis, water, or nutrient transport, suggesting that B-toxicity-induced water stress was responsible for root growth inhibition under B-stress. Recently, Tombuloglu et al. (2015) used high-throughput RNA-Seq to examine B-toxicity-induced alterations of gene profiles in barely and observed a total of 14,385 and 11,472 differentially expressed transcripts from high-B-treated roots and leaves, respectively, and concluded that genes involved in cell wall and cytoskeleton, plasma membrane, stress response, and signal transduction played key roles in the B-tolerance of barely. Although the transcriptional responses of herbaceous plants to B-toxicity have been investigated in some details, such data are very limited in woody plants.

In China, B-excess is observed in some citrus orchards. Li et al. (2015) measured the water-soluble B content of 319 soil samples and the B concentration of 319 leaf samples from 319 pummelo (*Citrus grandis*) orchards located in Pinghe, Zhangzhou, China. Up to 22.9 and 74.8% of orchards were excess in soil water-soluble B and leaf B, respectively. Therefore, it is very important to understand the molecular mechanisms on B-toxicity and B-tolerance of citrus. cDNA-amplified fragment length polymorphism (cDNA-AFLP), which does not need prior sequence information for the amplification, is a highly sensitive and reproducible approach for detecting polymorphisms in DNA. Therefore, cDNA-AFLP has been widely used for the identification of various stress-regulated genes and for the isolation of novel genes (Ditt et al., 2001; Fusco et al., 2005; Craciun et al., 2006; Paun and Schönswetter, 2012; Zhou et al., 2013; Vantini et al., 2015). Previously, we used cDNA-AFLP to assay long-term B-excess-responsive genes in B-tolerant *Citrus sinensis* and B-intolerant *C. grandis* leaves and obtained 132 and 68 differentially expressed genes from B-toxic *C. grandis* and *C. sinensis* leaves, respectively. Subsequent analysis suggested that the higher mRNA levels of genes involved in photosynthesis in B-toxic *C. sinensis* leaves were responsible for the higher CO_2_ assimilation, thus contributing to the B-tolerance of *C. sinensis*. Some genes related to stress responses also played a role in the B-tolerance of *C. sinensis* (Guo et al., 2014). In this study, we further examined long-term B-excess-induced alterations of gene profiles revealed by cDNA-AFLP in B-tolerant *C. sinensis* and B-intolerant *C. grandis* roots (Guo et al., 2014; Huang et al., 2014; Sang et al., 2015). The objectives were (a) to determine the differences in B-excess-responsive genes between roots and leaves; (b) to explore the mechanisms on B-toxicity and B-tolerance at translational level; and (c) to obtain B-excess-responsive genes possibly contributing to the B-tolerance of *C. sinensis*.

## Materials and methods

### Plant materials and experimental design

Boron-tolerant “Xuegan” (*C. sinensis*) and B-intolerant “Sour pummel” (*C. grandis*) seedlings were used in this study. Both seedling culture and B treatments were performed according to Guo et al. (2014). In a word, 13-week-old seedlings grown in 6 L pots containing sand in a greenhouse under natural photoperiod at Fujian Agriculture and Forestry University (FAFU), Fuzhou, China (26°5′ N, 119°14′ E), were irrigated every 2 days until dripping with nutrient solution containing 6 mM KNO_3_, 2 mM NH_4_H_2_PO_4_, 4 mM Ca(NO_3_)_2_, 1 mM MgSO_4_, 10 μM H_3_BO_3_, 2 μM ZnSO_4_, 2 μM MnCl_2_, 0.5 μM CuSO_4_, 0.065 μM (NH_4_)_6_Mo_7_O_24_ 20 μM Fe-EDTA, and 10 μM (control) or 400 μM (B-excess) H_3_BO_3_ for 15 weeks. Thereafter, about 5-mm long root tips from new white roots were excised and immediately immersed in liquid nitrogen, and then stored at −80°C until being used for the measurements of total soluble proteins and enzyme activities, and cDNA-AFLP and qRT-PCR analysis. The remaining seedlings were used to assay root B and phosphorus (P) concentrations.

### Measurements of root total soluble protein, B, and P concentrations

Root concentration of total soluble proteins was assayed according to Bradford (1976) after being extracted with 50 mM Na_2_HPO_4_–KH_2_PO_4_ (pH 7.0) and 5% (w/v) insoluble polyvinylpyrrolidone. There were five replicates per treatments.

For the measurements of root B and P concentrations, fibrous roots were harvested and dried at 70°C. Root B concentration was determined by ICP emission spectrometry after microwave digestion with HNO_3_ (Wang et al., 2006). Root P concentration was determined according to Ames (1966). There were four replicates per treatments.

### RNA extraction, cDNA synthesis, and cDNA-AFLP analysis

Equal amounts of frozen roots from five excess B-treated or control seedlings (one seedling per pot) of *C. grandis* or *C. sinensis* were mixed as a biological replicate. There were three biological replicates for each B treatment. Total RNA was extracted from ca. 300 mg of frozen roots using Recalcitrant Plant Total RNA Extraction Kit (Centrifugal column type, Bioteke Corporation, China). cDNA synthesis and cDNA-AFLP analysis were performed according to Guo et al. (2014) and Lu et al. (2015). Database resources of the National Center for Biotechnology Information (NCBI, http://www.ncbi.nlm.nih.gov) were used for the blast analysis of the differentially expressed transcript-derived fragments (TDFs) from excess B-treated *C. grandis* and *C. sinensis* roots. TDFs were blasted using BLASTX and BLASTN search engines (http://blast.ncbi.nlm.nih.gov/Blast.cgi), and a TDF was considered to be homologous when it had an *E* < 0.001 and a similarity ≥50%. Their functional categories were assigned based on the Gene Ontology (http://amigo1.geneontology.org/cgi-bin/amigo/go.cgi) and UniProt (http://www.uniprot.org/) database.

### qRT-PCR analysis

Root total RNA was extracted as described above. qRT-PCR analysis was carried out according to Zhou et al. (2013) and Lu et al. (2015). The sequences of the specific F and R primers designed from the sequences of 15 singleton TDFs using Primer Premier Version 5.0 (PREMIER Biosoft International, CA, USA) were summarized in Table S1. The samples subjected to qRT-PCR were performed in three biological replicates with two technical replicates. Each biological replicate was created by pooling equal roots from five seedlings (one seedling per pot). Citrus *actin* (GU911361.1) was used as an internal standard for the normalization of gene expression, and roots from control plants were used as a reference sample (set as 1).

### Analysis of root peroxidase, catalase, and lipoxygenase activities

Peroxidase (POD) and catalase (CAT) were extracted and assayed according to Li et al. (2010). Lipoxygenase (LOX) was assayed by the formation of conjugated dienes from linoleic acid according to Axelrod et al. (1981).

### Statistical analysis

Differences among four treatment combinations were analyzed by two (B levels) × two (species) ANOVA. Means were separated by the Duncan's new multiple range test at *P* < 0.05. Significant tests for two means (control and B-excess) were carried out by unpaired *t*-test at *P* < 0.05 level.

## Results

### Effects of B-excess on root B, P, and total soluble protein concentrations

**Figure 1:**
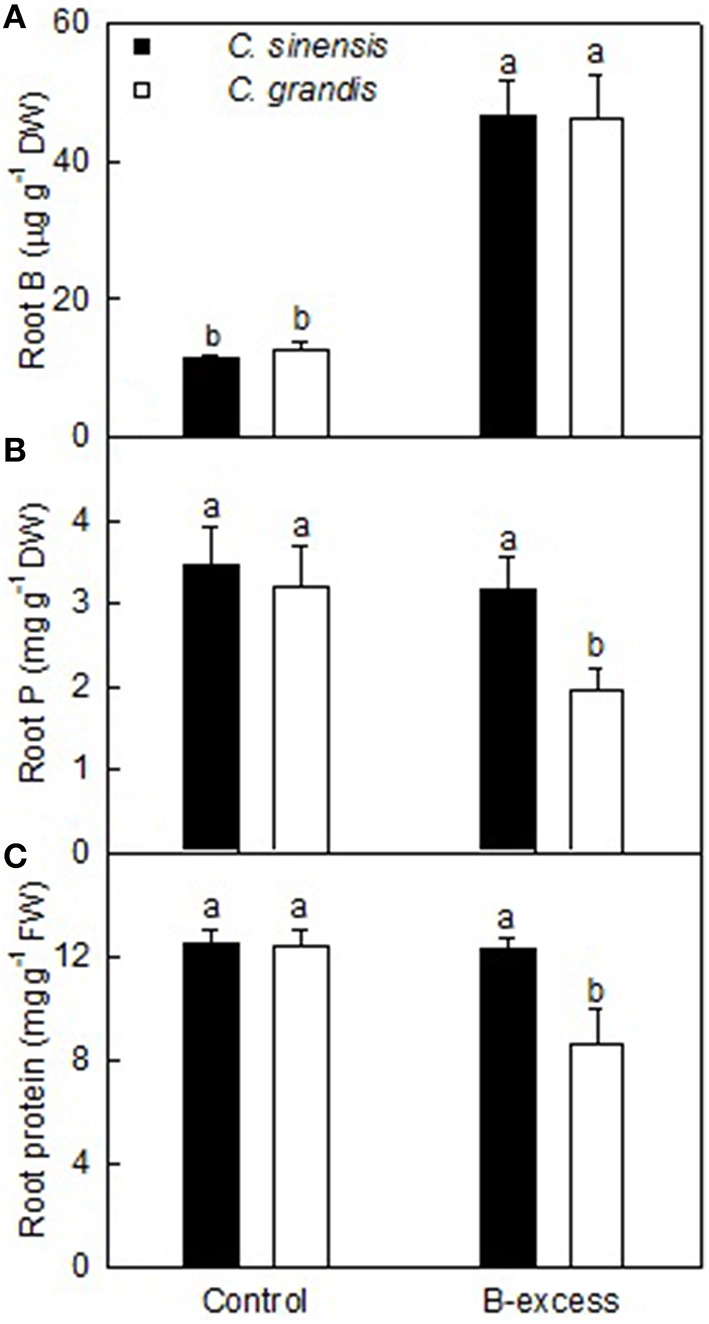
**Effects of B-excess on root B (A), P (B), and total soluble protein (C) concentrations**. Bars represent means ± *SD* (*n* = 4). Different letters above the bars indicate a significant difference at *P* < 0.05.

Root B concentration was higher in excess B-treated *C. grandis* and *C. sinensis* roots than in controls. Root B level did not differ significantly between the two citrus species at each given B treatment (Figure 1A).

Root P concentration did not significantly differ among four treatment combinations except for a drop in excess B-treated *C. grandis* roots (Figure 1B).

Boron excess only lowered total soluble protein concentration in *C. grandis* roots. Total soluble protein concentration was higher in excess B-treated *C. sinensis* roots than in excess B-treated *C. grandis* but was similar between control roots of the two citrus species (Figure 1C).

### Root B-excess-responsive genes revealed by cDNA-AFLP

**Figure 2:**
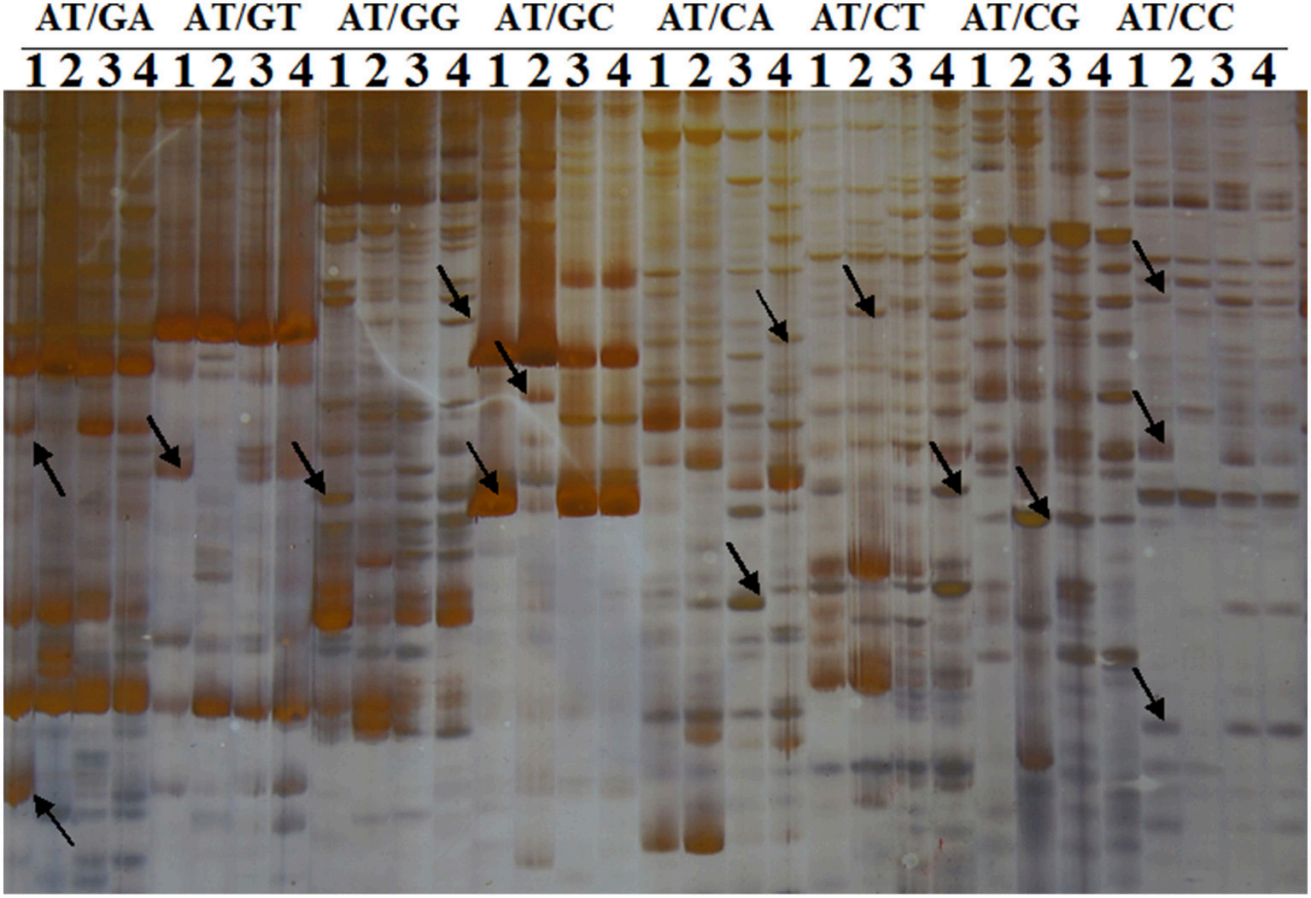
**cDNA-AFLP profiles using one ***Eco***R I selective primer and eight ***Mes*** I selective primers**. One *Eco*R I selective primer: *Eco*R I-AT; Eight *Mes* I selective primers: *Mes* I-GA, GA, GG, GC, CA, CT, CG, and CC. 1, Control roots of *C. grandis*; 2, Excess B-treated roots of *C. grandis*; 3, Control roots of *C. sinensis*; 4, Excess B-treated roots of *C. sinensis*. Arrows indicate differentially expressed TDFs.

A total of 256 selective primer combinations were employed to isolate B-excess-responsive genes from B-tolerant *C. sinensis* and B-intolerant *C. grandis* roots. A representative silver-stained cDNA-AFLP gel displaying the differentially expressed TDFs in excess B-treated *C. grandis* and *C. sinensis* roots was presented in Figure 2. As shown in Table 1, we obtained 5873 TDFs from control and B-toxic *C. sinensis* and *C. grandis* roots, 893 (589) of which were presented only in *C. grandis* (*C. sinensis*) roots and 4391 were shared by the both.

**Table 1:** **List of transcript-derived fragments (TDFs) isolated from control and boron (B)-toxic ***Citrus grandis*** and ***Citrus sinensis*** roots**.

	**Number of TDFs**			
	**Only present in *C. grandis***	**Only present in *C. sinensis***	**Present in both species**	**Total**
Total TDFs detected from gels	893	589	4391	5873
Total differentially expressed TDFs recovered from gels	156	82	56	294
TDFs produced useable sequence data	133	65	43	241
TDFs encoding known or putative proteins	68	37	18	123
TDFs encoding predicted, uncharacterized, hypothetical, or unnamed proteins	17	10	7	34
TDFs without database matches	48	18	18	84

**Figure 3:**
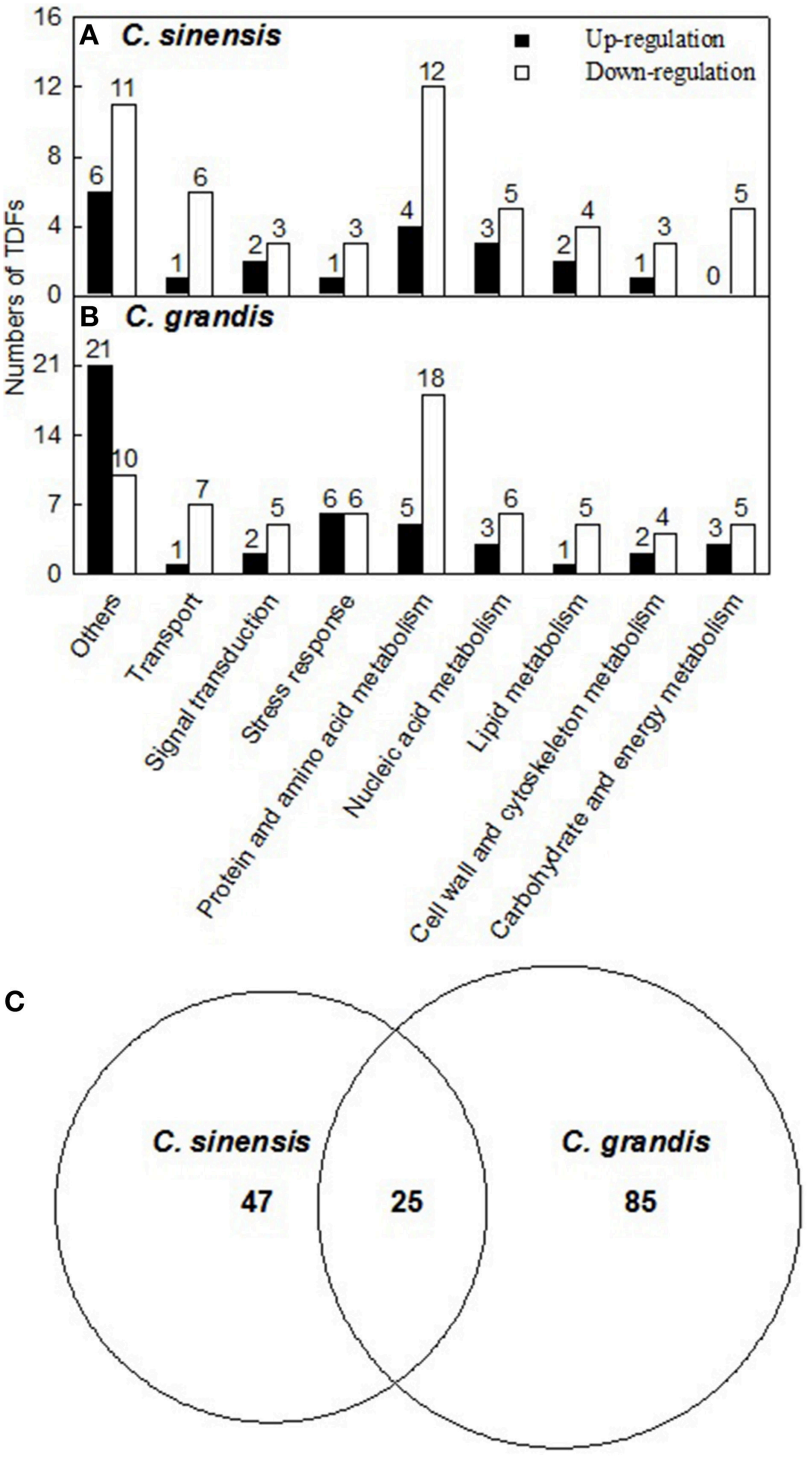
**Functional classification of differentially expressed TDFs under B-excess in ***Citrus grandis*** (A) and ***Citrus sinensis*** roots (B) and Venn diagram analysis of differentially expressed TDFs in citrus roots (C)**. Functional classification was performed based on the information reported for each sequence by The Gene Ontology (http://amigo1.geneontology. org/cgi-bin/amigo/go.cgi) and Uniprot (http://www.uniprot.org/).

A 1.5-fold cut-off was employed to determine up- and down-regulated TDFs, in addition to a *P*-value of < 0.05. According to the two criteria, we identified 294 B-excess-responsive TDFs from *C. grandis* and *C. sinensis* roots. After sequencing all these TDFs, we obtained readable sequences from 241 TDFs, 157 of which shared identity with genes encoding known, putative, uncharacterized, predicted, hypothetical, or unnamed proteins, and 84 showed no significant matches (Table 1). The 157 matched TDFs were related to carbohydrate and energy metabolism, cell wall and cytoskeleton metabolism, lipid metabolism, nucleic acid metabolism, protein and amino acid metabolism, stress response, signal transduction, transport, and others (Table 2 and Figures 3A,B). As shown in Tables 1, 2 and Figure 3C, most of these matched B-excess-responsive TDFs only presented in *C. sinensis* (47) or *C. grandis* (85) roots, only 25 TDFs were shared by the both. Obviously, long-term B-excess-induced alterations of gene profiles greatly differed between *C. sinensis* and *C. grandis* roots.

**Table 2:** **Homologies of differentially expressed cDNA-AFLP fragments with known gene sequences in database using BLASTN algorithm along their expression patterns in excess B-treated ***C. grandis*** (CG) and ***Citrus sinensis*** (CS) roots**.

**TDF #**	**Size (bp)**	**Homologies**	**Organism origin**	***E*-value**	**Similarity (%)**	**Sequence coverage (%)**	**Genebank ID**	**Ratio of BE/CK**	
								**CG**	**CS**
**CARBOHYDRATE AND ENERGY METABOLISM**									
232_1	286	Cytochrome c oxidase subunit Vb	*Dimocarpus longan*	7.00E-37	92	46	AEQ61828.1		0
101_5	179	NADH dehydrogenase subunit 4, partial (mitochondrion)	*Actinidia polygama*	1.00E-26	96	9	CAD60445.1		0
41_2	315	Electron transfer flavoprotein-ubiquinone oxidoreductase	*Medicago truncatula*	2.00E-51	80	17	XP_003597528.1		0.41
98_2	254	Pyruvate dehydrogenase E1 alpha subunit	*Arabidopsis thaliana*	1.00E-26	83	16	NP_171617.1		0.32
**53_4**	**251**	**Pyruvate kinase**	***Arabidopsis thaliana***	**5.00E-39**	**96**	**12**	**NP_001078275.1**	**0**	**0.35**
185_1	241	Phosphoglycerate kinase	*Arabidopsis thaliana*	3.00E-15	51	14	NP_178073.1	0	
150_1	391	Alternative oxidase	*Mangifera indica*	7.00E-33	87	21	CAA55892.1	0	
137_2	204	Cytochrome P450	*Arabidopsis thaliana*	2.00E-19	66	11	AAB67854.1	0.29	
9_2	201	Ferredoxin-NADP reductase, root isozyme 2	*Arabidopsis thaliana*	8.00E-30	86	15	NP_973942.1	2.25	
26_2	314	Dephospho-CoA kinase	*Medicago truncatula*	5.00E-44	73	26	XP_003625901.1	2.83	
248_1	261	Sucrose synthase	*Citrus unshiu*	2.00E-44	100	7	BAA89049.1	0.27	
116_2	353	NADP-dependent D-sorbitol-6-phosphate dehydrogenase	*Medicago truncatula*	2.00E-39	86	27	XP_003607080.1	2.43	
**CELL WALL AND CYTOSKELETON METABOLISM**									
65_1	305	Root hair defective 3 GTP-binding protein (RHD3)	*Arabidopsis thaliana*	4.00E-25	84	13	NP_177439.2		+
151_2	303	Alpha-1,4-glucan-protein synthase [UDP-forming], putative	*Ricinus communis*	3.00E-60	92	30	XP_002525910.1		0.34
37_2	240	Fucosyltransferase 8	*Arabidopsis thaliana*	2.00E-21	63	15	NP_001077531.1		0
99_2	254	Polygalacturonase-like protein	*Arabidopsis thaliana*	3.00E-11	54	17	BAA95779.1		0
50_1	255	Villin 4	*Arabidopsis thaliana*	2.00E-17	71	6	NP_194745.1	0	
26_1	385	O-methyltransferase	*Citrus sinensis x Citrus reticulata*	1.00E-41	65	36	ABP94018.1	0	
231_3	219	Beta-D-xylosidase 4	*Arabidopsis thaliana*	7.00E-29	87	9	NP_201262.1	0.46	
138_1	415	Cell wall-associated hydrolase, partial	*Medicago truncatula*	5.00E-44	96	19	XP_003637074.1	+	
249_1	384	Protein SPIRAL1-like2	*Arabidopsis thaliana*	3.00E-27	80	62	NP_177083.1	3.72	
253_1	346	S-acyltransferase	*Arabidopsis lyrata subsp.lyrata*	5.00E-39	76	15	XP_002887235.1	0.51	
**LIPID METABOLISM**									
183_2	380	Glycerol-3-phosphate acyltransferase 9	*Arabidopsis thaliana*	9.00E-62	82	28	NP_568925.1	0.29	
44_1	95	Dihydroxyacetone kinase	*Arabidopsis thaliana*	4.00E-05	84	4	NP_188404.1	+	
249_2	330	Coproporphyrinogen III oxidase	*Arabidopsis thaliana*	3.00E-55	88	23	NP_171847.4	0.33	
24_2	328	Cinnamate 4-hydroxylase	*Citrus x paradisi*	7.00E-27	100	19	AAK57011.1	0.41	
53_3	284	Lipoxygenase	*Vitis vinifera*	9.00E-41	80	10	ACZ17393.1	0	
**129_3**	**290**	**Fatty acid/sphingolipid desaturase**	***Arabidopsis thaliana***	**3.00E-40**	**80**	**17**	**NP_182144.1**	**0**	**0**
150_2	379	Acyl carrier protein	*Camellia oleifera*	2.00E-16	87	53	AFK31314.1		0
89_2	295	Acyl carrier protein 3	*Helianthus annuus*	1.00E-30	81	70	ADV16367.1		0
9_1	213	Citrus dioxygenase	*Citrus limetta*	6.00E-36	94	20	AER36089.1		+
42_1	261	1,4-dihydroxy-2-naphthoyl-CoA thioesterase 1	*Arabidopsis thaliana*	2.00E-31	81	32	NP_175266.1		3.61
103_6	152	JAZ3	*Vitis rupestris*	2.00E-11	70	13	AEP60134.1		0.24
**NUCLEIC ACID METABOLISM**									
140_2	249	Histone deacetylase 19	*Arabidopsis thaliana*	6.00E-30	81	13	NP_001078511.1		2.7
129_1	299	Histone H4	*Arabidopsis thaliana*	1.00E-32	100	45	NP_001077477.1		2.2
148_1	208	Zinc finger protein CONSTANS-LIKE 5-like	*Glycine max*	5.00E-25	87	18	NP_001239972.1		+
82_1	213	DNA binding protein	*Arabidopsis lyrata subsp. Lyrata*	2.00E-04	74	5	XP_002889547.1		0.5
28_1	308	PREDICTED: ATP-dependent DNA helicase recG-like	*Vitis vinifera*	6.00E-15	53	10	XP_002280664.2		0
131_4	296	Transcription initiation factor IIB-2	*Arabidopsis thaliana*	2.00E-51	93	23	NP_187644.1		0.36
60_1	395	Poly(A)-binding protein	*Daucus carota*	5.00E-24	80	16	AAK30205.1		0
**246_1**	**309**	**Transcription factor TCP15**	***Arabidopsis thaliana***	**1.00E-41**	**89**	**19**	**NP_564973.1**	**0**	**0**
79_1	272	KNAT4 homeobox protein	*Arabidopsis thaliana*	4.00E-47	99	18	CAA63131.1	+	
123_2	287	DEAD-box ATP-dependent RNA helicase 20	*Arabidopsis thaliana*	5.00E-30	77	16	NP_175911.1	4.1	
195_1	268	Ribonucleotide reductase RNR1 like protein, partial	*Arabidopsis thaliana*	1.00E-46	94	27	CAA69026.1	0	
133_4	273	Putative phosphoribosylaminoimidazole carboxylase/AIR carboxylase	*Arabidopsis thaliana*	1.00E-40	80	11	NP_181305.2	0	
134_1	308	Polypyrimidine tract-binding protein 1	*Arabidopsis thaliana*	9.00E-53	90	20	NP_186764.1	0	
142_1	231	Transcription factor/ transcription initiation factor	*Medicago truncatula*	7.00E-24	74	13	XP_003597404.1	0.53	
252_1	310	AT1G56110, partial	*Arabidopsis thaliana*	5.00E-24	74	23	BAH20197.1	0.33	
81_3	325	Hypothetical protein POPTR_0001s24860g	*Populus trichocarpa*	2.00E-49	79	14	XP_002299866.1	1.84	
**PROTEIN AND ACID METABOLISM**									
85_2	356	Ubiquitin-protein ligase 1	*Arabidopsis thaliana*	4.00E-59	88%	3%	AAF36454.1		0.38
**132_3**	**384**	**E3 ubiquitin-protein ligase MARCH3**	***Medicago truncatula***	**6.00E-09**	**86**	**27**	**XP_003613768.1**	**0**	**0**
**73_2**	**324**	**E3 ubiquitin-protein ligase ATL6**	***Arabidopsis thaliana***	**8.00E-17**	**50**	**23**	**AAD33584.1**	**0**	**0**
42_3	180	Ubiquitin, partial	*Oryza sativa*	2.00E-26	96	46	AAL77200.1	+	
86_1	398	Ubiquitin-protein ligase, putative	*Ricinus communis*	8.00E-25	57	18	XP_002520193.1	0.43	
112_1	461	E3 ubiquitin-protein ligase UPL5	*Medicago truncatula*	3.00E-42	70	18	XP_003594229.1	0	
27_1	333	ARM repeat superfamily protein	*Arabidopsis thaliana*	3.00E-52	83	6	NP_198149.2	0	
230_1	258	Aspartic protease, partial	*Dimocarpus longan*	1.00E-05	71	26	AEJ76922.1		+
**62_1**	**326**	**Aspartic proteinase nepenthesin-1**	***Medicago truncatula***	**1.00E-37**	**77**	**19**	**XP_003627883.1**	**0**	**0**
61_1	335	Protein ASPARTIC PROTEASE IN GUARD CELL 1	*Arabidopsis thaliana*	3.00E-40	65	18	NP_188478.1	1.79	
118_5	208	Serine carboxypeptidase II-3	*Medicago truncatula*	3.00E-22	72	14	XP_003592243.1	0.21	
41_1	141	Drought-inducible cysteine proteinase RD19A precursor	*Arabidopsis thaliana*	5.00E-16	86	35	BAD94010.1	0	
24_1	352	Gamma-glutamyl hydrolase 2	*Arabidopsis thaliana*	2.00E-34	74	25	NP_565186.2	0.35	
253_4	264	Protein disulfide isomerase	*Zea mays*	7.00E-23	64	13	ACG47473.1	0.43	
184_1	267	Protein disulfide isomerase-like 1-1	*Arabidopsis thaliana*	2.00E-09	56	17	NP_849696.1		0.38
12_1	361	Elongation factor 1-alpha, partial	*Arabidopsis thaliana*	2.00E-21	93	60	BAD94755.1		2.04
121_2	160	40S ribosomal protein S8	*Zea mays*	3.00E-19	98	19	NP_001105391.1		2.52
48_1	303	40S ribosomal protein S4	*Medicago truncatula*	2.00E-52	90	24	XP_003604568.1		0.44
246_5	228	40S ribosomal protein S2	*Arabidopsis lyrata subsp. Lyrata*	5.00E-29	87	27	XP_002878157.1		0
**9_3**	**198**	**60S ribosomal protein L7a**	***Zea mays***	**2.00E-15**	**91**	**19**	**ACG47588.1**	+	+
**153_2**	**261**	**40S ribosomal protein S5**	***Capsicum annuum***	**4.00E-44**	**100**	**31**	**AAR89617.1**	**0**	**0.39**
**80_2**	**320**	**50S ribosomal protein L2, chloroplastic**	***Citrus sinensis***	**3.00E-63**	**99**	**62**	**YP_740517.1**	**0**	**0**
64_2	291	60S ribosomal protein L10-1	*Arabidopsis thaliana*	2.00E-54	90	30	NP_563945.2	+	
140_1	335	40S ribosomal protein S29	*Zea mays*	9.00E-26	86	74	ACG30830.1	3.12	
38_1	290	60S ribosomal protein L15-1	*Arabidopsis thaliana*	2.00E-47	84	33	NP_193405.1	0.41	
14_2	225	Translation initiation factor eIF-4A1	*Arabidopsis thaliana*	1.00E-37	100	13	CAC43286.1	0	
**229_1**	**327**	**Methionine synthase 2**	***Arabidopsis thaliana***	**3.00E-59**	**92**	**12**	**NP_187028.1**	**0**	**0.46**
**144_2**	**266**	**Threonyl-tRNA synthetase**	***Medicago truncatula***	**2.00E-41**	**88**	**11**	**XP_003601575.1**	**0**	**0.22**
**5_1**	**354**	**S-adenosyl-L-homocystein hydrolase**	***Gossypium hirsutum***	**2.00E-27**	**89**	**19**	**ACJ11250.1**	**0**	**0**
229_2	192	Bifunctional aminoacyl-tRNA synthetase, putative, expressed	*Oryza sativa Japonica Group*	5.00E-30	96	10	ABA97740.1	0.34	
**STRESS RESPONSE**									
**182_3**	**214**	**Homogentisate phytyltransferase 1**	***Arabidopsis thaliana***	**3.00E-14**	**51**	**12**	**NP_849984.1**	+	+
64_1	309	Obg-like ATPase 1	*Arabidopsis thaliana*	5.00E-51	87	20	NP_174346.1	0	
99_3	238	Peroxidase	*Medicago truncatula*	3.00E-22	60	14	XP_003615990.1	0	
**231_5**	**188**	**Catalase, partial**	***Citrus maxima***	**9.00E-31**	**91**	**19**	**ACY30463.1**	**0**	**0**
30_1	320	DJ-1 family protein	*Arabidopsis lyrata subsp.lyrata*	9.00E-28	73	24	XP_002894433.1	0.24	
233_5	141	Heat shock protein 70	*Nicotiana benthamiana*	3.00E-05	75	33	BAD02271.1	+	
237_1	305	BAG family molecular chaperone regulator 7	*Arabidopsis thaliana*	4.00E-14	67	16	NP_201045.1	0.35	
131_1	332	Putative senescence-associated protein, partial	*Trichosanthes dioica*	5.00E-05	100	100	ABN50029.1	+	
17_1	296	Putative senescence-associated protein, partial	*Ipomoea ni*	6.00E-52	93	25	BAF46313.1	2.52	
104_3	171	WD repeat phosphoinositide-interacting-like protein	*Medicago truncatula*	4.00E-21	96	11	XP_003591137.1	3.15	
77_5	112	Universal stress protein A-like protein	*Medicago truncatula*	2.00E-09	74	12	XP_003616191.1		0.37
140_3	220	ACR toxin-sensitivity inducing protein (mitochondrion)	*Citrus jambhiri*	4.00E-10	96	10	BAB85481.1	+	
251_5	266	Ankyrin-like protein	*Arabidopsis thaliana*	7.00E-38	82	9	BAB10271.1		0
146_5	285	Leucine-rich repeat containing protein, putative	*Ricinus communis*	1.00E-20	59	8	XP_002523984.1	0	
**SIGNAL TRANSDUCTION**									
35_1	341	ATP/GTP/Ca++ binding protein	*Cucumis melo subsp.melo*	2.00E-44	74	18	ABR67417.1		3.04
80_3	194	Calcium-dependent lipid-binding domain-containing protein	*Arabidopsis thaliana*	2.00E-19	80	13	NP_564576.1	0.41	
67_2	232	Calcium-binding EF-hand-containing protein	*Arabidopsis thaliana*	2.00E-07	52	5	NP_173582.2	0.49	
35_2	267	Calcium ion binding protein, putative	*Ricinus communis*	7.00E-32	67	18	XP_002534034.1	0.48	
103_2	207	Serine/threonine-protein kinase	*Nicotiana attenuata*	3.00E-20	71	8	AEI84329.1		+
**251_3**	**301**	**Leucine-rich repeat receptor-like protein kinase**	***Medicago truncatula***	**4.00E-34**	**68**	**15**	**XP_003600547.1**	**0**	**0**
53_2	350	ATP binding/kinase/protein serine/threonine kinase	*Vitis vinifera*	3.00E-50	80	12	CAQ58615.1	+	
122_1	343	Receptor-like protein kinase-like protein	*Glycine max*	4.00E-46	77	13	ACM89521.1	0	
103_3	207	14-3-3 protein	*Litchi chinensis*	6.00E-33	95	19	ADP00759.1	+	
117_1	184	Auxin-responsive protein	*Gossypium hirsutum*	1.00E-07	54	15	AEE25651.1		0
39_1	330	Phytochrome A, partial	*Populus tremula*	2.00E-51	86	7	AEK26583.1		0
**TRANSPORT**									
**251_2**	**316**	**H**+**-ATPase 4, plasma membrane-type**	***Arabidopsis thaliana***	**3.00E-23**	**92**	**10**	**NP_190378.2**	+	+
**76_3**	**270**	**Exocyst complex component 84B**	***Arabidopsis thaliana***	**8.00E-25**	**64**	**9**	**NP_199794.1**	**0**	**0**
77_8	448	Vacuolar membrane ATPase subunit c”	*Citrus limon*	1.00E-71	99	50	AAO73433.1		0
151_1	336	Exocyst subunit exo70 family protein B1	*Arabidopsis thaliana*	1.00E-50	82	15	NP_200651.1		0
135_1	326	ADP ribosylation factor	*Medicago truncatula*	2.00E-50	100	88	XP_003591354.1		0.49
246_4	228	Peroxin 7	*Arabidopsis thaliana*	5.00E-07	75	16	NP_174220.1		0.45
39_2	216	Protein transport protein SEC61 gamma subunit	*Medicago truncatula*	3.00E-04	90	64	XP_003616767.1		0
183_3	236	ABC transporter I family member 20	*Arabidopsis thaliana*	1.00E-12	69	20	NP_195847.1	0.42	
253_2	318	Metal tolerance protein	*Carica papaya*	7.00E-52	89	26	ADI24923.1	0	
19_1	316	Sec61 transport protein	*Populus trichocarpa*	6.00E-54	88	16	XP_002331716.1	0.5	
141_2	272	Synaptobrevin-like protein	*Medicago truncatula*	6.00E-35	83	20	XP_003617238.1	0	
79_3	196	PRA1 (prenylated RAB acceptor) family protein	*Medicago truncatula*	1.00E-18	65	18	XP_003597623.1	0.31	
10_1	147	Protein tolB	*Medicago truncatula*	1.00E-09	72	7	XP_003637684.1	0.37	
**OTHERS**									
98_3	331	Rubber elongation factor protein	*Arabidopsis thaliana*	2.00E-23	63	31	NP_187201.1	+	
133_5	259	FRIGIDA-like protein	*Arabidopsis thaliana*	9.00E-25	69	12	NP_850923.1	0	
**121_1**	**248**	**Limonoid UDP-glucosyltransferase**	***Citrus maxima***	**5.00E-11**	**70**	**16**	**ABY27084.1**	**0**	**0**
77_4	318	ATP sulfurylase 1	*Arabidopsis thaliana*	2.00E-54	97	18	NP_188929.1	0	
38_2	134	FAD-binding berberine family protein	*Medicago truncatula*	2.00E-13	85	6	XP_003594602.1	0.41	
189_1	309	Probable methyltransferase PMT14	*Arabidopsis thaliana*	4.00E-37	62	15	BAH19630.1		+
41_3	398	1-deoxy-D-xylulose-5-phosphate synthase, partial	*Bixa orellana*	2.00E-65	87	68	AAU29063.1	1.9	
173_1	286	DPP6 N-terminal domain-like protein	*Arabidopsis thaliana*	4.00E-21	70	12	NP_564147.1	0.3	
66_1	284	F22G5.28	*Arabidopsis thaliana*	6.00E-36	68	12	AAF79556.1	+	
147_4	254	PREDICTED: uncharacterized protein LOC100264520	*Vitis vinifera*	3.00E-38	84	24	XP_002276822.1		0.51
109_3	277	Unnamed protein product, partial	*Vitis vinifera*	1.00E-19	64	50	CBI31118.3	0.28	
153_3	226	Predicted protein	*Arabidopsis lyrata subsp*.	2.00E-16	69	46	XP_002871569.1	1.78	
22_4	287	Hypothetical protein VITISV_014691	*Vitis vinifera*	2.00E-27	65	21	CAN71367.1	2.32	
158_1	177	PREDICTED: uncharacterized protein LOC100255693	*Vitis vinifera*	5.00E-09	51	13	XP_002279857.1	1.33	
21_1	231	AT5G10200-like protein	*Arabidopsis arenosa*	1.00E-31	90	9	ACK44505.1		+
163_3	228	PREDICTED: uncharacterized protein LOC100262433	*Vitis vinifera*	2.00E-30	77	11	XP_002284886.1	0	
27_2	267	Predicted protein	*Populus trichocarpa*	5.00E-27	63	30	XP_002330640.1	0	
116_1	401	Hypothetical protein MTR_1g005920	*Medicago truncatula*	3.00E-25	79	75	XP_003588318.1		0.39
**163_2**	**330**	**Unnamed protein product**	***Vitis vinifera***	**8.00E-07**	**69**	**6**	**CBI37596.3**	+	**0**
170_2	212	Predicted protein	*Populus trichocarpa*	7.00E-07	68	14	XP_002309501.1		0
77_6	230	Hypothetical protein MTR_5g051130	*Medicago truncatula*	4.00E-11	79	6	XP_003614394.1		0.51
105_2	277	PREDICTED: UPF0197 transmembrane protein C11orf10	*Glycine max*	3.00E-25	87	42	XP_003549239.1	2.13	
**108_2**	**144**	**Hypothetical protein SORBIDRAFT_0351s002020**	***Sorghum bicolor***	**7.00E-16**	**100**	**28**	**XP_002489033.1**	+	**0**
109_5	178	Unknown	*Glycine max*	6.00E-15	89	57	ACU14517.1	+	
118_3	287	Hypothetical protein MTR_5g051140	*Medicago truncatula*	1.00E-14	93	8	XP_003614395.1		2.47
**170_1**	**296**	**Unknown**	***Glycine max***	**5.00E-31**	**95**	**30**	**ACU24256.1**	**1.8**	**2.11**
173_3	213	Hypothetical protein MTR_5g051120	*Medicago truncatula*	1.00E-25	88	18	XP_003614393.1	1.73	
**178_1**	**306**	**Hypothetical protein**	***Lilium longiflorum***	**2.00E-25**	**96**	**100**	**ABO20854.1**	+	**0**
193_3	244	Hypothetical protein MTR_4g091430	*Medicago truncatula*	3.00E-11	57	48	XP_003608262.1	+	
**228_1**	**306**	**Hypothetical protein**	***Arabidopsis thaliana***	**1.00E-39**	**100**	**11**	**BAF01964.1**	**1.32**	+
237_3	204	PREDICTED: uncharacterized protein LOC100800109	*Glycine max*	2.00E-31	92	54	XP_003541224.1		+
249_5	224	Hypothetical protein	*Zea mays*	4.00E-20	98	24	ACG27665.1	1.38	
**37_3**	**217**	**Hypothetical protein SORBIDRAFT_0070s002020**	***Sorghum bicolor***	**3.00E-23**	**82**	**72**	**XP_002489102.1**	**3.01**	**0.39**
53_5	165	Hypothetical protein SORBIDRAFT_1994s002010	*Sorghum bicolor*	2.00E-10	100	28	XP_002488913.1	2.73	
6_3	218	Hypothetical protein MTR_5g051030	*Medicago truncatula*	8.00E-25	95	9	XP_003614386.1	2.06	
85_1	369	Hypothetical protein SORBIDRAFT_1368s002010	*Sorghum bicolor*	7.00E-52	91	66	XP_002488947.1		0.47
94_1	222	Hypothetical protein MTR_8g040260	*Medicago truncatula*	2.00E-06	51	31	XP_003627937.1	+	
146_4	245	Putative uncharacterized protein Sb0351s002020	*Sorghum bicolor*	3.00E-12	97	47	XP_002489033.1	0.54	
252_4	190	Conserved hypothetical protein	*Ricinus communis*	2.00E-09	83	19	XP_002532022.1	0.38	
**133_7**	**217**	**Hypothetical protein SORBIDRAFT_0227s002010**	***Sorghum bicolor***	**2.00E-15**	**97**	**48**	**XP_002489051.1**	+	**0.41**

*Data are means of three replicates; Expression ratio, + indicates that TDFs were only isolated from excess B-treated roots. #, Number; BE, B-excess; CK, Control. Functional classification was performed based on the information reported for each sequence by The Gene Ontology (http://amigo1.geneontology.org/cgi-bin/amigo/go.cgi) and Uniprot (http://www.uniprot.org/). Ratio of BE/CK was obtained by gel image analysis performed with PDQuest version 8.0.1 (Bio-Rad, Hercules, CA, USA). B-excess-responsive TDFs shared by the two citrus species were highlighted in bold*.

### qRT-PCR validation of root differentially expressed TDFs revealed by cDNA-AFLP

**Figure 4:**
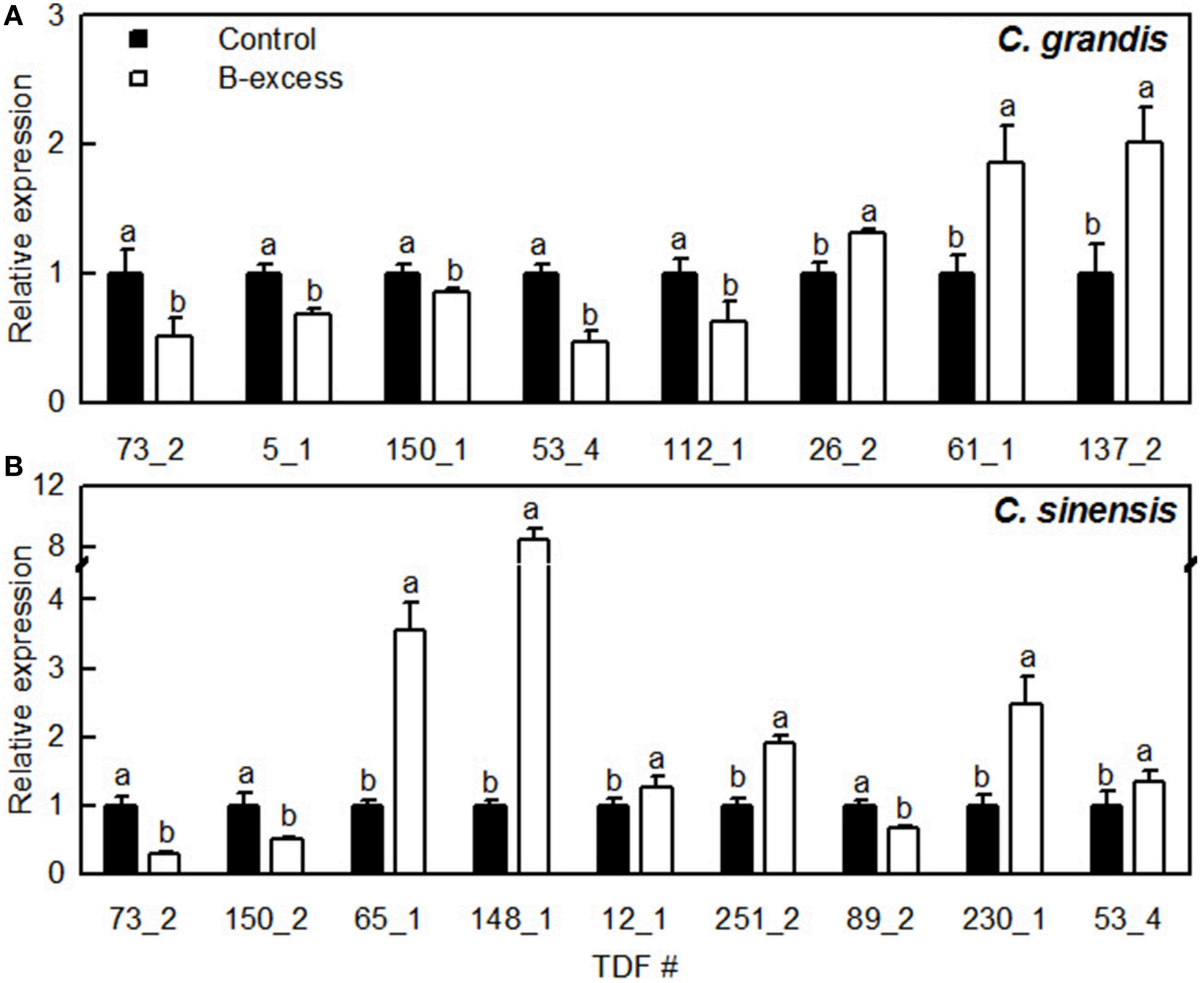
**Relative expression levels of TDFs from ***Citrus grandis*** (A) and ***Citrus sinensis*** (B) roots. qRT-PCR was run in three biological replicates with two technical replicates**. Citrus *actin* (GU911361.1) was employed as an internal standard, and roots from control plants were used as a reference sample (set as 1). Bars represent means ± *SD*. Different letters above the bars indicate a significant difference at *P* < 0.05.

As shown in Figure 4, we selected eight and nine TDFs from *C. grandis* and *C. sinensis* roots, respectively, for qRT-PCR analysis in order to examine their expression profiles obtained by cDNA-AFLP. Except for TDFs #137_2 and 53_4 in *C. grandis* roots, the expression patterns of the other TDFs produced by qRT-PCR agreed with the data obtained by cDNA-AFLP.

### Analysis of enzymes for three differentially expressed TDFs

**Figure 5:**
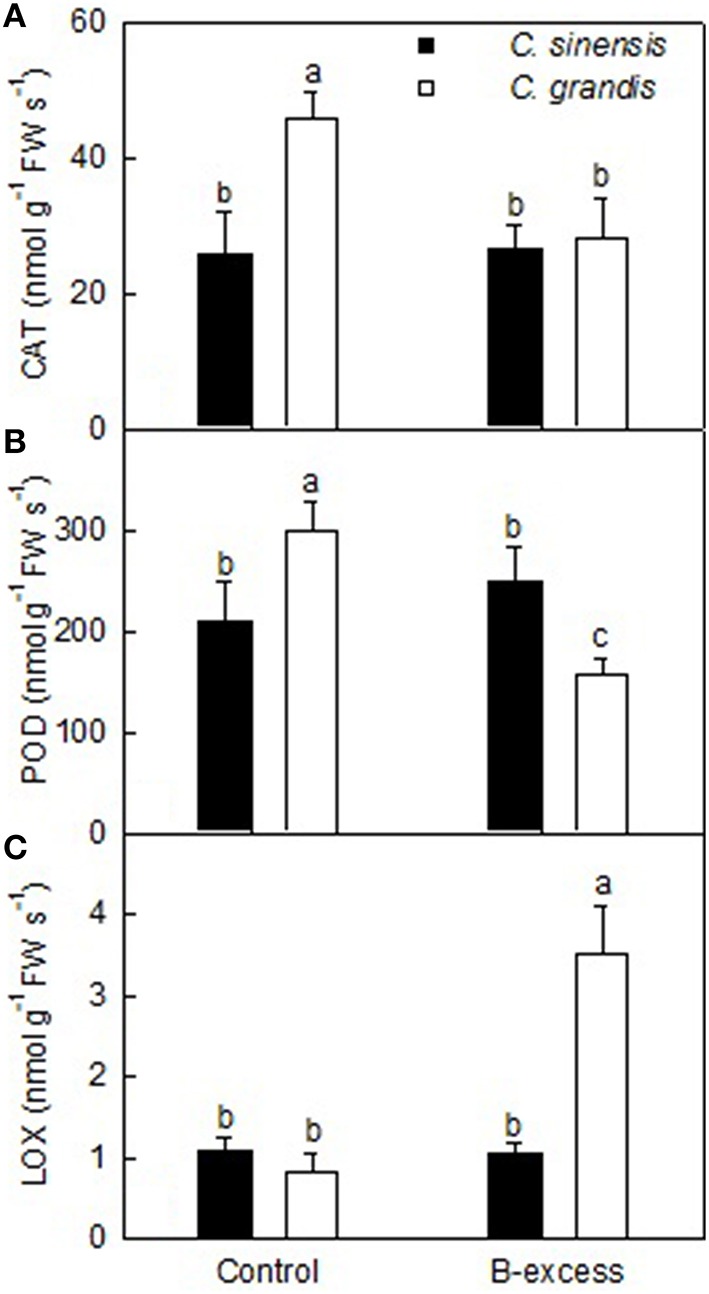
**Effects of B-excess on root CAT (A), POD (B) and LOX (C) activities**. Bars represent means ± *SD* (*n* = 4). Different letters above the bars indicate a significant difference at *P* < 0.05.

We assayed the activities of enzymes for three B-excess-responsive TDFs in *C. sinensis* and *C. grandis* roots. The enzyme activities did not match fully with the gene expression levels (Table 2 and Figure 5). The discrepancy between the both means that post-modifications (PTMs) might influence these enzyme abundances and their activities. In addition, all these enzymes are composed of two or more isoenzymes, which might be responsible for the discrepancy between gene expression levels and enzyme activities.

## Discussion

We isolated more B-excess-responsive TDFs from *C. grandis* roots than from *C. sinensis*, demonstrating that B-excess affected gene expression more in B-intolerant *C. grandis* roots than in B-tolerant *C. sinensis* roots (Tables 1, 2). This agrees with our data that B-excess only lowered P and total soluble protein concentrations in *C. grandis* roots (Figures 1B,C) and also agrees with our report that more B-excess-responsive TDFs were obtained from *C. grandis* leaves than from *C. sinensis* leaves (Guo et al., 2014). Despite this, great differences existed in B-excess-induced alterations of gene profiles between roots and leaves. For example, most of B-excess-responsive TDFs were identified only from roots or leaves, and only three TDFs with the same genebank ID (i.e., BAD94010.1, NP_564576.1, and XP_003614394.1) were isolated from roots and leaves of *C. sinensis* and *C. grandis*. Moreover, the responses of the three TDFs to B-excess differed between leaves and roots (Table 2; Guo et al., 2014).

### Effects of B-excess on root carbohydrate and energy metabolism

As shown in Table 2, the mRNA levels of three TDFs encoding cytochrome *c* oxidase subunit Vb (TDF #231_1), NADH dehydrogenase subunit 4 (TDF #101_5), and electron transfer flavoprotein-ubiquinone oxidoreductase (TDF #41_2), the components of the mitochondrial respiratory chain, and of two TDFs encoding pyruvate dehydrogenase E1 α subunit (TDF #98_2) and PK (TDF #53_4) involved in glycolysis were lowered in excess B-treated *C. sinensis* roots, implying that respiration was impaired in these roots. Similarly, both *PK* (TDF #53_4) and *phosphoglycerate kinase* (PGK, TDF #185-1) related to glycolysis were inhibited in excess B-treated *C. grandis* roots. Interestingly, the expression of alternative oxidase (AOX, a cyanide-insensitive terminal oxidase) gene (TDF #150_1) was also down-regulated in excess B-treated *C. grandis* roots. Unlike the cytochrome pathway, the AOX pathway, which is cyanide-insensitive and can operate when the cytochrome pathway is restricted, plays a role in inhibiting ROS production in plant mitochondria (Popov et al., 1997; Purvis, 1997; Maxwell et al., 1999; Zidenga et al., 2012). Cytochrome P450s (CytoP450s), a superfamily of monooxygenases, have a key role in (a)biotic stresses. We found that *CytoP45*0 (TDF #137_2) was repressed in excess B-treated *C. grandis* roots, as obtained on B-toxic *Arabidopsis* roots (Aquea et al., 2012). Transgenic potato and tobacco plants expressing *CytoP450* displayed increased monooxygenase activity and enhanced tolerance to oxidative stress after herbicide treatment (Gorinova et al., 2005). Therefore, B-excess-induced down-regulation of *AOX* and *CytoP450* in *C. grandis* roots might contribute to the less B-tolerance of *C. grandis*.

### Effects of B-excess on root cell wall and cytoskeleton metabolism

Cell wall and cytoskeleton metabolism were altered in excess B-treated *C. sinensis* and *C. grandis* roots, as indicated by B-excess-induced alterations of 10 related genes (i.e., 65_1, 151_2, 37_2, 99_2, 50_1, 26_1, 231_3, 138_1, 249_1, and 253_1; Table 2). Similar results have been obtained on excess B-treated citrus leaves (Guo et al., 2014; Sang et al., 2015). This agrees with the reports that B-toxicity affected citrus root cell walls (Huang et al., 2014) and that B-toxicity disturbed actin filament organization, thus impairing the directional transport of cell wall materials and cell wall construction in *Malus domestica* pollen tubes (Fang et al., 2016). *Root Hair Defective 3* (*RHD3*), a gene encoding a putative GTP-binding protein, is required for actin organization, cell wall biosynthesis, and normal cell expansion in *Arabidopsis* roots (Wang et al., 2002; Hu et al., 2003). Overexpression of *PeRHD* in poplar led to less formation of adventitious roots, better-developed lateral roots, and more and longer root hairs (Xu et al., 2012). B-excess-induced up-regulation of *RHD3* in *C. sinensis* roots might be an adaptive response by maintaining root normal growth and development. By contrast, *villin 4* (TDF #50_1), an actin filament bundling protein gene, was down-regulated in excess B-treated *C. grandis* roots (Table 2). Zhang et al. (2011) demonstrated that *Arabidopsi*s *villin 4* was required for normal root hair growth through regulating the organization of actin bundles. The down-regulation of *villin 4* implied that B-excess impaired actin organization in *C. grandis* roots, thus affecting the root growth. The differences in Al-induced alterations of root cell wall and cytoskeleton metabolism between the two citrus species might be responsible for the greater decrease in root dry weight in B-toxic *C. grandis* seedlings relative to B-toxic *C. sinensis* ones (Guo et al., 2014).

### Effects of B-excess on root lipid metabolism

We found that all these differentially expressed TDFs (i.e., TDFs #183_2, 249_2, 24_2, 53_3, 129_3, 150_2, 89_2, and 103_6) related to lipid metabolism were inhibited in excess B-treated *C. grandis* and *C. sinensis* roots except for three TDFs (i.e., TDFs #44_1, 9_1, and 42_1; Table 2), implying that lipid metabolism pathway in these roots was upset. Glycerol-3-phosphate acyltransferase (GPAT), the first enzyme for the biosynthesis of glycerolipids in all organisms, plays crucial roles in basic cellular metabolism. AtGPAT9 is required for the biosynthesis of membrane lipids and oils. A knockout mutant of *AtGPAT9* was homozygous lethal (Shockey et al., 2016). We found that *GPAT9* (TDF #183_2) was down-regulated in excess B-treated *C. grandis* roots (Table 2), suggesting that B-toxicity impaired membrane lipid biosynthesis, thus lowering B-tolerance of *C. grandis*.

Lipoxygenase (LOX) reactions may initiate the generation of ROS, thus leading to the loss of membranes (Pérez-Gilabert et al., 2001). Inal et al. (2009) found that B-excess increased malondialdehyde (MDA) level and LOX activity, and silicon (Si) enhanced B-tolerance by decreasing B-excess-induced increases in MDA level and LOX activity in barely. B-excess down-regulated (did not significantly affect) *LOX* expression in *C. grandis* (*C. sinensis*) roots (Table 2) but increased (did not significantly affect) LOX activity in *C. grandis* (*C. sinensis*) roots (Figure 5C). Therefore, the observed higher LOX activity might lower the B-tolerance of *C. grandis*.

We found that *citrus dioxygenase* (TDF #9_1) was up-regulated in excess B-treated *C. sinensis* roots (Table 2). Alpha-dioxygenase (α-DOX) functions in protecting tissues against oxidative damage and cell death (Tirajoh et al., 2005). Therefore, *citrus dioxygenase* might be involved in the B-tolerance of *C. sinensis*.

### Effects of B-excess on root nucleic acid metabolism

Boron-toxicity affects nucleic acid metabolic process in higher plants (Kasajima and Fujiwara, 2007; Sakamoto et al., 2011; Guo et al., 2014). As expected, three up- (i.e., TDFs #140_2, 129_1 and 148_1) and five down-regulated (i.e., TDFs #82_1, 28_1, 131_4, 60_1 and 246_1) TDFs, and three up- (i.e., TDFs #79_1, 123_2 and 81_3) and six down-regulated (TDFs #246_1, 195_1, 133_4, 134_1, 142_1, and 252_1) TDFs related to nucleic acid metabolism were isolated from excess B-treated *C. sinensis* and *C. grandis* roots, respectively (Table 2). Histone deacetylases and histone acetyltransferases, which catalyze histone acetylation and deacetylation, respectively, play a key role in the regulation of gene and genome activity. *Arabidopsis* histone deacetylase 19 (AtHDA19 or AtHD1) plays a role in plant growth and development. Reduction in *AtHDA19* expression by T-DNA insertion or antisense inhibition in *Arabidopsis* caused various developmental abnormalities such as suppression of apical dominance, early senescence, and flower defects (Tian and Chen, 2001; Tian et al., 2003). Recently, Chen et al. (2015) obtained a total of 86 P-deficiency-responsive genes, which showed lower expression in AtHDA19-RNAi *Arabidopsis* plants relative to 35S:AtHDA19 lines. Kaya et al. (2009) observed that high-B-treated tomato leaves had lower P concentration, and that supplementary P alleviated high-B-induced decreases in plant growth and fruit yield. B-toxicity decreased P uptake in wheat (Singh et al., 1990). Therefore, excess B-induced up-regulated of *HDA19* (TDF #140_2) in *C. sinensis* roots (Table 2) might be an adaptive response by the maintenances of P homeostasis. This agrees with our report that B-toxicity only reduced P level in *C. grandis* leaves (Guo et al., 2014) and roots (Figure 1B). Similarly, histone H4 (TDF #129_1) was up-regulated in excess B-treated *C. sinensis* roots (Table 2), which agrees with the reports that *histone H4* was induced in Al-toxic rice roots (Mao et al., 2004) and Al-toxicity increased B level and decreased P level in plant roots, stems, and leaves (Jiang et al., 2009a,b). Histones H2A, H2B, H3, and H4, the core component of nucleosome, play crucial roles in chromosomal stability, gene regulation, and DNA repair and replication (Turner, 2002; Drury et al., 2012; Zhu et al., 2012). Sakamoto et al. (2011) demonstrated that plant condensin II played a crucial role in the tolerance of *Arabidopsis* to B-toxicity by alleviating DNA damage. Therefore, the up-regulation of *histone H4* in excess B-treated *C. sinensis* roots (Table 2) might contribute to the B-tolerance of *C. sinensis* by preventing genome from damage resulting from B-excess.

Ribonucleotide reductase (RNR), an enzyme comprising two R1 and two R2, plays a key role in DNA repair and replication (Chabouté et al., 1998; Wang and Liu, 2006). The down of *RNR1 like protein* (TDF #195_1) in excess B-treated *C. grandis* roots (Table 2) implied that B-toxicity impaired DNA repair and replication, thus lowering the B-tolerance of *C. grandis*. Similarly, the expression levels of *phosphoribosylaminoimidazole carboxylase/AIR carboxylase* (TDF #133_4) involved in nucleotide biosynthesis were down-regulated in excess B-treated *C. grandis* roots.

### Effects of B-excess on root protein and amino acid metabolism

As shown in Table 2, we isolated 10 down-regulated (i.e., TDFs #85_2, 132_3, 73_2, 86_1, 112_1, 27_1, 62_1, 118_5, 41_1, and 24_1) and three up-regulated (i.e., TDFs #42_3, 230_1, and 61_1) TDFs involved in protein degradation, two down-regulated TDFs (i.e., TDFs #253_4 and 184_1,) involved in protein folding and stability, five up-regulated (i.e., TDFs #12_1, 121_2, 9_3, 64_2, and 140_1) and six down-regulated (i.e., TDFs #48_1, 246_5, 153_2, 80_2, 38_1, and 14_2) TDFs involved in protein biosynthesis, and four down-regulated TDFs (i.e., TDFs #229_1, 144_2, 5_1, and 229_2) involved in amino acid metabolism. Obviously, protein and amino acid metabolism was impaired in B-toxic citrus roots.

### Effects of B-excess on root stress-related gene expression

Boron-excess causes the accumulation of ROS, thus resulting in oxidative and membrane damage in plant roots and leaves (Cervilla et al., 2007; Pandey and Archana, 2013; Martínez-Cuenca et al., 2015). An antioxidant response has been suggested to abate B-toxic damage in some higher plants (Gunes et al., 2006). Here, we isolated one up-regulated *homogentisate phytyltransferase 1* (*HPT1*; TDF #182_3) from excess B-treated *C. grandis* and *C. sinensis* roots (Table 2). HPT catalyzes the committed step in the biosynthesis of tocopherol (vitamin E). Collakova and DellaPenna (2003) showed that transgenic *Arabidopsis* plants overexpressing *HPT1* had an elevated total tocopherol level in seed and leaves. The up-regulation of *HPT1* agrees with the increased requirement for the detoxification of ROS in excess B-treated *C. grandis* and *C. sinensis* roots. In addition, *Obg-like ATPase 1* (*YchF1*; TDF #64_1), a negative regulator of the antioxidant response (Cheung et al., 2013), was down-regulated in excess B-treated *C. grandis* roots. However, *POD* (TDF #99_3) in *C. grandis* roots and *CAT* (TDF #231_5) in *C. grandis* and *C. sinensis* roots were down-regulated by B-excess. Enzyme activity analysis showed that B-excess lowered CAT and POD activities in *C. grandis* roots but did not significantly affect their activities in *C. sinensis* roots (Figures 5A,B). Besides, the expression level of an antioxidant protein gene (Xu and Møller, 2010; Xu et al., 2010), *DJ-1 family protein* (TDF #30_1) was reduced in excess B-treated *C. grandis* roots (Table 2). Obviously, excess B-treated *C. sinensis* roots kept higher antioxidant capacity than excess B-treated *C. grandis* roots.

Two putative senescence-associated protein genes (i.e., TDFs #131_1 and 17_1) were induced by B-excess in *C. grandis* roots (Table 2), suggesting that senescence was accelerated in these roots. This agrees with our report that B-excess affected root growth more in *C. grandis* than in *C. sinensis* seedlings (Guo et al., 2014). Similarly, autophagic cell death might be accelerated in excess B-treated *C. grandis* roots, as indicated by the enhanced expression level of *WD repeat phosphoinositide-interacting-like protein* (*WILP*; TDF #104_3) in these roots (Table 2). This agrees with our report that B-excess led to cell death in *C. grandis* roots (Huang et al., 2014).

### Effects of B-excess on root signal transduction

Calcium (Ca) signaling plays vital roles in plant responses to various stresses (Knight, 2000). Ca can alleviate B-toxicity and high-B inhibits Ca uptake in higher plants (Singh et al., 1990; Turan et al., 2009; Siddiqui et al., 2013). Hence, genes related to Ca^2+^ signal transduction should be altered in excess B-treated citrus roots. As expected, we isolated one up-regulated TDF: *ATP/GTP/Ca*^2+^ *binding protein* [also called as *mitochondrial Rho GTPase* (*MIRO*)] from B-toxic *C. sinensis* roots (Table 2). Jayasekaran et al. (2006) found that the transcript level of *MIRO2* was elevated in ABA- and salt-treated *Arabidopsis* plants, and that the knock-out mutants of *MIRO2* were less tolerance to ABA, salt, and osmotic treatments than the wild-type. By contrast, we only isolated three down-regulated TDFs (i.e., TDFs #80_3, 67-2, and 35_2) involved in Ca signals from B-toxic *C. grandis* roots. Therefore, Ca^2+^-mediated signal played a role in the tolerance of citrus plants to B-toxicity.

### Effects of B-excess on transport processes in root cells

As shown in Table 2, the mRNA levels of all the 13 differentially expressed TDFs related to cellular transport were reduced in excess B-treated *C. grandis* and *C. sinensis* roots except for one up-regulated *H*^+^*-ATPase 4* (*AHA4*, TDF #251_2), indicating that the transport of some substances (i.e., ion and proteins) was altered in these roots. This disagrees with our report that most of the differentially expressed genes involved in cellular transport were up-regulated in excess B-treated *C. grandis* leaves and three up- and three down-regulated genes were isolated from excess B-treated *C. sinensis* leaves (Guo et al., 2014).

*ABC transporter I family member 20* (*ABCI20*; TDF #183_2) was down-regulated in excess B-treated *C. grandis* roots (Table 2). This agrees with our report that *ABC1 family protein* (AT5g24810) was repressed in high-B-treated *C. grandis* leaves (Guo et al., 2014). In eukaryotic organisms, ABC transporters have been shown to play a role in the efflux (extracellular or intracellular into the vacuoles) of potentially toxic compounds (i.e., heavy metals, alkaloids, and organic anions; Gaedeke et al., 2001). Transgenic *Arabidopsis* plants overexpressing *AtPDR8* encoding an ABC transporter displayed higher Cd- and Pb-tolerance accompanied by less accumulation of Cd in the shoots or roots. By contrast, mutants were less tolerance to both the metals accompanied by more accumulation of Cd (Kim et al., 2007). Therefore, the down-regulation of *ABCI20* and *ABC1 family protein* in roots and leaves might reduce the efflux of B from the PM, thus increasing free B level and lowering *C. grandis* B-tolerance. Interestingly, middle leaves from excess B-treated *C. grandis* seedlings displayed more free B and less bound B than those from *C. sinensis*, but free B and bound B in excess B-treated roots did not significantly differ between the two citrus species (Huang et al., 2014). Previous studies showed that *C. sinensis* had defective nodulin-26-like intrinsic protein 2 (NIP2) gene, which is involved in influx of Si, B, and other metalloids (Schnurbusch et al., 2010; Deshmukh et al., 2015). Therefore, NIP2 could be involved in compartmentation of B differently among this species. However, B-excess did not alter *NIP2* expression in citrus roots, suggesting that other transporters played a role in citrus compartmentation of B or that *NIP2* was regulated at the post-translational level.

## Conclusions

We used cDNA-AFLP to investigate comparatively the effects of B-excess on gene profiles in B-tolerant *C. sinensis* and B-intolerant *C. grandis* roots, and isolated 20 up- and 52 down-regulated, and 44 up- and 66 down-regulated TDFs from excess B-treated *C. sinensis* and *C. grandis* roots, respectively, thereby demonstrating that gene expression was less affected in the former than in the latter. Besides, B-excess only lowered P and total soluble protein concentrations in *C. grandis* roots. Obviously, *C. sinensis* seedlings had higher B-tolerance than *C. grandis* ones. Our results suggested that the following several aspects led to the difference in the B-tolerance between the both, including: (a) *AOX* and *CytoP450* were down-regulated only in excess B-treated *C. grandis* roots; (b) B-excess up-regulated *RHD3* in *C. sinensis* roots and down-regulated *villin4* in *C. grandis* roots, and accordingly, root dry weight was less reduced by B-excess in the former; (c) antioxidant systems were impaired in excess B-treated *C. grandis* roots, hence accelerating root senescence; (d) TDFs involved in Ca^2+^ signals were down-regulated in excess B-treated *C. grandis* roots and up-regulated in excess B-treated *C. sinensis* roots. In addition, B-excess-responsive genes related to lipid (i.e., *GPAT9* and *citrus dioxygenase*) and nucleic acid (i.e., *histone deacetylase 19, histone 4*, and *RNR1 like protein*) metabolisms also possibly contributed to the difference in the B-tolerance between the both. Our data provided some new clues for our understanding of the molecular mechanisms on citrus B-toxicity and B-tolerance.

## Author contributions

PG carried out most of the experiments and drafted the manuscript. YQ participated in the design of the study. LY participated in the design of the study and coordination. XY participated in the measurement of B and P. JH participated in the statistical analysis. LC designed and directed the study and revised the manuscript. All authors have read and approved the final manuscript.

## Funding

Our work was funded by the earmarked fund for China Agriculture Research System (No. CARS-27).

### Conflict of interest statement

The authors declare that the research was conducted in the absence of any commercial or financial relationships that could be construed as a potential conflict of interest.

## References

[B1] Ames B. N. (1966). Assay of inorganic phosphate, total phosphate and phosphatase. Meth. Enzymol. 8, 115–118. 10.1016/0076-6879(66)08014-5

[B2] Aquea F. Federici F. Moscoso C. Vega A. Jullian P. Haseloff J.. (2012). A molecular framework for the inhibition of *Arabidopsis* root growth in response to boron toxicity. Plant Cell Environ. 35, 719–734. 10.1111/j.1365-3040.2011.02446.x 21988710

[B3] Axelrod B. Cheesbrough T. M. Laakso S. (1981). Lipoxygenase from soybeans. Meth. Enzymol. 71, 441–451. 10.1016/0076-6879(81)71055-3

[B4] Ayvaz M. Guven A. Fagerstedt K. (2015). Does excess boron affect hormone levels of potato cultivars? Biotechnol. Biotec. Eq. 29, 887–891. 10.1080/13102818.2015.1053411

[B5] Ayvaz M. Koyuncu M. Guven A. Fagerstedt K. V. (2012). Does boron affect hormone levels of barley cultivars? Eur. Asian J. Biosci. 6, 113–120. 10.5053/ejobios.2012.6.0.14

[B6] Bradford M. M. (1976). A rapid and sensitive method for quantitation of microgram quantities of protein utilizing the principle of protein-dye binding. Anal. Biochem. 72, 248–254. 10.1016/0003-2697(76)90527-3 942051

[B7] Cervilla L. M. Blasco B. Ríos J. Romero L. Ruiz J. (2007). Oxidative stress and antioxidants in tomato (*Solanum lycopericum*) plants subjected to boron toxicity. Ann. Bot. 100, 747–756. 10.1093/aob/mcm156 17660516 PMC2749626

[B8] Cervilla L. M. Blasco B. Ríos J. J. Rosales M. A. Rubio-Wilhelmi M. M. Sánchez-Rodríguez E.. (2009). Response of nitrogen metabolism to boron toxicity in tomato plants. Plant Biol. 11, 671–677. 10.1111/j.1438-8677.2008.00167.x 19689774

[B9] Chabouté M. E. Combettes B. Clément B. Gigot C. Philipps G. (1998). Molecular characterization of tobacco ribonucleotide reductase RNR1 and RNR2 cDNAs and cell cycle-regulated expression in synchronized plant cells. Plant Mol. Biol. 38, 797–806. 10.1023/A:1006083318906 9862497

[B10] Chen C. Y. Wu K. Schmidt W. (2015). The histone deacetylase HDA19 controls root cell elongation and modulates a subset of phosphate starvation responses in *Arabidopsis*. Sci. Rep. 5:15708. 10.1038/srep15708 26508133 PMC4623716

[B11] Chen L. S. Han S. Qi Y. P. Yang L. T. (2012). Boron stresses and tolerance in citrus. Afr. J. Biotech. 11, 5961–5969. 10.5897/AJBX11.073

[B12] Chen M. Mishra S. Heckathorn S. A. Frantz J. M. Krause C. (2014). Proteomic analysis of *Arabidopsis thaliana* leaves in response to acute boron deficiency and toxicity reveals effects on photosynthesis, carbohydrate metabolism, and protein synthesis. J. Plant Physiol. 171, 235–242. 10.1016/j.jplph.2013.07.008 23988561

[B13] Cheung M. Y. Li M. W. Yung Y. L. Wen C. Q. Lam H. M. (2013). The unconventional P-loop NTPase OsYchF1 and its regulator OsGAP1 play opposite roles in salinity stress tolerance. Plant Cell Environ. 36, 2008–2020. 10.1111/pce.12108 23550829

[B14] Collakova E. DellaPenna D. (2003). Homogentisate phytyltransferase activity is limiting for tocopherol biosynthesis in *Arabidopsis*. Plant Physiol. 131, 632–642. 10.1104/pp.015222 12586887 PMC166839

[B15] Craciun A. R. Courbot M. Bourgis F. Salis P. Saumitou-Laprade P. Verbruggen N. (2006). Comparative cDNA-AFLP analysis of Cd-tolerant and -sensitive genotypes derived from crosses between the Cd hyperaccumulator *Arabidopsis halleri* and *Arabidopsis lyrata* ssp. petraea. J. Exp. Bot. 57, 2967–2983. 10.1093/jxb/erl062 16916885

[B16] Deshmukh R. K. Vivancos J. Ramakrishnan G. Guérin V. Carpentier G. Sonah H.. (2015). A precise spacing between the NPA domains of aquaporins is essential for silicon permeability in plants. Plant J. 83, 489–500. 10.1111/tpj.12904 26095507

[B17] Ditt R. F. Nester E. W. Comai L. (2001). Plant gene expression response to *Agrobacterium tumefaciens*. Proc. Natl. Acad. Sci. U.S.A. 98, 10954–10959. 10.1073/pnas.191383498 11535836 PMC58580

[B18] Drury G. E. Dowle A. A. Ashford D. A. Waterworth W. M. Thomas J. West C. E. (2012). Dynamics of plant histone modifications in response to DNA damage. Biochem. J. 445, 393–401. 10.1042/BJ20111956 22574698

[B19] Fang K. Zhang W. Xing Y. Zhang Q. Yang L. Cao Q.. (2016). Boron toxicity causes multiple effects on *Malus domestica* pollen tube growth. Front. Plant Sci. 7:208. 10.3389/fpls.2016.00208 26955377 PMC4768074

[B20] Fusco N. Micheletto L. DalCorso G. Borgato L. Furini A. (2005). Identification of cadmium-regulated genes by cDNA-AFLP in the heavy metal accumulator *Brassica juncea* L. J. Exp. Bot. 56, 3017–3027. 10.1093/jxb/eri299 16216843

[B21] Gaedeke N. Klein M. Kolukisaoglu U. Forestier C. Müller A. Ansorge M.. (2001). The *Arabidopsis thaliana* ABC transporter AtMRP5 controls root development and stomata movement. EMBO J. 20, 1875–1887. 10.1093/emboj/20.8.1875 11296221 PMC125232

[B22] Gorinova N. Nedkovska M. Atanassov A. (2005). Cytochrome P450 monooxygenases as a tool for metabolization herbicides in plants. Biotechnol. Biotechnol. Equip. 19, 105–115. Special Issue. 10.1080/13102818.2005.10817290

[B23] Gunes A. Soylemezoglu G. Inal A. Bagci E. G. Coban S. Sahin O. (2006). Antioxidant and stomatal responses of grapevine (*Vitis vinifera* L.) to boron toxicity. Sci. Hort. 110, 279–284. 10.1016/j.scienta.2006.07.014

[B24] Guo P. Qi Y. P. Yang L. T. Ye X. Jiang H. X. Huang J. H.. (2014). cDNA-AFLP analysis reveals the adaptive responses of citrus to long-term boron-toxicity. BMC Plant Biol. 14:284. 10.1186/s12870-014-0284-5 25348611 PMC4219002

[B25] Han S. Tang N. Jiang H. X. Yang L. T. Li Y. Chen L. S. (2009). CO_2_ assimilation, photosystem II photochemistry, carbohydrate metabolism and antioxidant system of citrus leaves in response to boron stress. Plant Sci. 176, 143–153. 10.1016/j.plantsci.2008.10.004

[B26] Hassan M. Oldach K. Baumann U. Langridge P. Sutton T. (2010). Genes mapping to boron tolerance QTL in barley identified by suppression subtractive hybridization. Plant Cell Environ. 33, 188–198. 10.1111/j.1365-3040.2009.02069.x 19906153

[B27] Hu Y. Zhang R. G. Morriison W. H. 3rd Ye Z. H. (2003). The *Arabidopsis RHD3* gene is required for cell wall biosynthesis and actin organization. Planta 217, 912–921. 10.1007/s00425-003-1067-7 12844267

[B28] Huang J. H. Cai Z. J. Wen S. X. Guo P. Ye X. Lin G. Z. (2014). Effects of boron toxicity on root and leaf anatomy in two citrus species differing in boron tolerance. Trees Struct. Funct. 28, 1653–1666. 10.1007/s00468-014-1075-1

[B29] Huang J. H. Qi Y. P. Wen S. X. Guo P. Chen X. M. Chen L. S. (2016). Illumina microRNA profiles reveal the involvement of miR397a in citrus adaptation to long-term boron toxicity via modulating secondary cell-wall biosynthesis. Sci. Rep. 6:22900. 10.1038/srep22900 26962011 PMC4790630

[B30] Inal A. Pilbeam D. J. Gunes A. (2009). Silicon increases tolerance to boron toxicity and reduces oxidative damage in barley. J. Plant Nutr. 32, 112–128. 10.1080/01904160802533767

[B31] Jayasekaran K. Kim K. N. Vivekanandan M. Shin J. S. Ok S. H. (2006). Novel calcium-binding GTPase (AtCBG) involved in ABA-mediated salt stress signaling in *Arabidopsis*. Plant Cell Rep. 25, 1255–1262. 10.1007/s00299-006-0195-5 16832621

[B32] Jiang H. X. Tang N. Zheng J. G. Chen L. S. (2009b). Antagonistic actions of boron against inhibitory effects of aluminum toxicity on growth, CO_2_ assimilation, ribulose-1,5-bisphosphate carboxylase/oxygenase, and photosynthetic electron transport probed by the JIP-test, of *Citrus grandis* seedlings. BMC Plant Biol. 9:102. 10.1186/1471-2229-9-102 19646270 PMC2731759

[B33] Jiang H. X. Tang N. Zheng J. G. Li Y. Chen L. S. (2009a). Phosphorus alleviates aluminum-induced inhibition of growth and photosynthesis in *Citrus grandis* seedlings. Physiol. Plant. 137, 298–311. 10.1111/j.1399-3054.2009.01288.x 19832942

[B34] Kasajima I. Fujiwara T. (2007). Identification of novel *Arabidopsis thaliana* genes which are induced by high levels of boron. Plant Biotechnol. 24, 355–360. 10.5511/plantbiotechnology.24.355

[B35] Kaya C. Tuna A. L. Dikilitas M. Ashraf M. Koskeroglu S. Guneri M. (2009). Supplementary phosphorus can alleviate boron toxicity in tomato. Sci. Hort. 121, 284–288. 10.1016/j.scienta.2009.02.011

[B36] Keles Y. Oncel I. Yenice N. (2004). Relationship between boron content and antioxidant compounds in citrus leaves taken from fields with different water sources. Plant Soil 265, 343–353. 10.1007/s11104-005-0646-8

[B37] Kim D. Y. Bovet L. Maeshima M. Martinoia E. Lee Y. (2007). The ABC transporter AtPDR8 is a cadmium extrusion pump conferring heavy metal resistance. Plant J. 50, 207–218. 10.1111/j.1365-313X.2007.03044.x 17355438

[B38] Knight H. (2000). Calcium signaling during abiotic stress in plants. Int. Rev. Cytol. 195, 269–324. 10.1016/S0074-7696(08)62707-2 10603578

[B39] Li Q. Chen L. S. Jiang H. X. Tang N. Yang L. T. Lin Z. H.. (2010). Effects of manganese-excess on CO_2_ assimilation, ribulose-1,5-bisphosphate carboxylase/oxygenase, carbohydrates and photosynthetic electron transport of leaves, and antioxidant systems of leaves and roots in *Citrus grandis* seedlings. BMC Plant Biol. 10:42. 10.1186/1471-2229-10-42 20205939 PMC2848762

[B40] Li Y. Han M. Q. Lin F. Ten Y. Lin J. Zhu D. H. (2015). Soil chemical properties, ‘Guanximiyou’ pummelo leaf mineral nutrient status and fruit quality in the southern region of Fujian province, China. J. Soil Sci. Plant Nutr. 15, 615–628. 10.4067/s0718-95162015005000029

[B41] Lu Y. B. Qi Y. P. Lee J. Guo P. Ye X. Jia M.. (2015). Long-term boron-deficiency-responsive genes revealed by cDNA-AFLP differ between *Citrus sinensis* roots and leaves. Front. Plant Sci. 6:585. 10.3389/fpls.2015.00585 26284101 PMC4517394

[B42] Mao C. H. Yi K. Yang L. Zheng B. Wu Y. Liu F. (2004). Identification of aluminium regulated genes by cDNA-AFLP in rice (*Oryza sativa* L.): aluminium-regulated genes for the metabolism of cell wall component. J. Exp. Bot. 55, 137–143. 10.1093/jxb/erh030 14645395

[B43] Martínez-Cuenca M. R. Martínez-Alcántara B. Quiñones A. Ruiz M. Iglesias D. J. Primo-Millo E.. (2015). Physiological and molecular responses to excess boron in *Citrus macrophylla* W. PLoS ONE 10:e0134372. 10.1371/journal.pone.0134372 26225859 PMC4520451

[B44] Maxwell D. P. Wang Y. McIntosh L. (1999). The alternative oxidase lowers mitochondrial reactive oxygen production in plant cells. Proc. Natl. Acad. Sci. U.S.A. 96, 8271–8276. 10.1073/pnas.96.14.8271 10393984 PMC22224

[B45] Mesquita G. L. Zambrosi F. C. B. Tanaka F. A. O. Boaretto R. M. Quaggio J. A. Ribeiro R. V.. (2016). Anatomical and physiological responses of citrus trees to varying boron availability are dependent on rootstock. Front. Plant Sci. 7:224. 10.3389/fpls.2016.00224 26973670 PMC4777737

[B46] Nable R. O. Banuelos G. S. Paull J. G. (1997). Boron toxicity. Plant Soil 193, 181–198. 10.1023/A:1004272227886

[B47] Padmanabhan P. Babaoğlu M. Terry N. (2012). A comparative transcriptomic analysis of the extremely boron tolerant plant Puccinellia distans with the moderately boron tolerant *Gypsophila arrostil*. Plant Cell Rep. 31, 1407–1413. 10.1007/s00299-012-1256-6 22484861

[B48] Pandey N. Archana (2013). Antioxidant responses and water status in *Brassica* seedlings subjected to boron stress. Acta Physiol. Plant. 35, 697–706. 10.1007/s11738-012-1110-z

[B49] Papadakis I. E. Dimassi K. N. Bosabalidis A. M. Therios I. N. Patakas A. Giannakoula A. (2004a). Effects of B excess on some physiological and anatomical parameters of ‘Navelina’ orange plants grafted on two rootstocks. Environ. Exp. Bot. 51, 247–257. 10.1016/j.envexpbot.2003.11.004

[B50] Papadakis I. E. Dimassi K. N. Bosabalidis A. M. Therios I. N. Patakas A. Giannakoula A. (2004b). Boron toxicity in ‘Clementine’ mandarin plants grafted on two rootstocks. Plant Sci. 166, 539–547. 10.1016/j.plantsci.2003.10.027

[B51] Papadakis I. E. Dimassi K. N. Therios I. N. (2003). Response of two citrus genotypes to six boron concentrations: concentration and distribution of nutrients, total absorption, and nutrient use efficiency. Aust. J. Agr. Res. 54, 571–580. 10.1071/AR02163

[B52] Paun O. Schönswetter P. (2012). Amplified fragment length polymorphism: an invaluable fingerprinting technique for genomic, transcriptomic, and epigenetic studies. Methods Mol. Biol. 862, 75–87. 10.1007/978-1-61779-609-8_7 22419490 PMC3513352

[B53] Pérez-Gilabert M. López-Nicolás J. M. Carmona F. G. (2001). Purification of a novel lipoxygenase from eggplant (*Solanum melongena*) fruit chloroplast. Physiol. Plant. 111, 276–282. 10.1034/j.1399-3054.2001.1110303.x 11240910

[B54] Popov V. N. Simonian R. A. Skulachev V. P. Starkov A. A. (1997). Inhibition of the alternative oxidase stimulates H_2_O_2_ production in plant mitochondria. FEBS Lett. 415, 87–90. 10.1016/S0014-5793(97)01099-5 9326375

[B55] Purvis A. C. (1997). Role of the alternative oxidase in limiting superoxide production in plant mitochondria. Physiol. Plant. 100, 165–170. 10.1111/j.1399-3054.1997.tb03468.x

[B56] Reid R. J. Hayes J. E. Post A. Stangoulis J. C. R. Graham R. D. (2004). A critical analysis of the causes of boron toxicity in plants. Plant Cell Environ. 27, 1405–1414. 10.1111/j.1365-3040.2004.01243.x

[B57] Sakamoto T. Inui Y. T. Uraguchi S. Yoshizumi T. Matsunaga S. Mastui M.. (2011). Condensin II alleviates DNA damage and is essential for tolerance of boron overload stress in *Arabidopsis*. Plant Cell 23, 3533–3546. 10.1105/tpc.111.086314 21917552 PMC3203421

[B58] Sang W. Huang Z. R. Qi Y. P. Yang L. T. Guo P. Chen L. S. (2015). An investigation of boron-toxicity in leaves of two citrus species differing in boron-tolerance using comparative proteomics. J. Proteomics 123, 128–146. 10.1016/j.jprot.2015.04.007 25892131

[B59] Schnurbusch T. Hayes J. Hrmova M. Baumann U. Ramesh S. A. Tyerman S. D.. (2010). Boron toxicity tolerance in barley through reduced expression of the multifunctional aquaporin HvNIP2;1. Plant Physiol. 153, 1706–1715. 10.1104/pp.110.158832 20581256 PMC2923888

[B60] Sheng O. Zhou G. F. Wei Q. J. Peng S. A. Deng X. X. (2010). Effects of excess boron on growth, gas exchange, and boron status of four orange scion-rootstock combinations. J. Plant. Nutr. Soil Sci. 173, 469–476. 10.1002/jpln.200800273

[B61] Shockey J. Regmi A. Cotton K. Adhikari N. Browse J. Bates P. D. (2016). Identification of *Arabidopsis GPAT9* (At5g60620) as an essential gene involved in triacylglycerol biosynthesis. Plant Physiol. 170, 163–179. 10.1104/pp.15.01563 26586834 PMC4704598

[B62] Siddiqui M. H. Al-Whaibi M. H. Sakran A. M. Ali H. M. Basalah M. O. Faisal M. (2013). Calcium-induced amelioration of boron toxicity in radish. J. Plant Growth Regul. 32, 61–71. 10.1007/s00344-012-9276-6

[B63] Singh J. P. Dahiya D. J. Narwal R. P. (1990). Boron uptake and toxicity in wheat in relation to zinc supply. Fert. Res. 24, 105–110. 10.1007/BF01073228

[B64] Tian L. Chen Z. J. (2001). Blocking histone deacetylation in *Arabidopsis* induces pleiotropic effects on plant gene regulation and development. Proc. Natl. Acad. Sci. U.S.A. 98, 200–205. 10.1073/pnas.98.1.200 11134508 PMC14568

[B65] Tian L. Wang J. Fong M. P. Chen M. Cao H. Gelvin S. B.. (2003). Genetic control of developmental changes induced by disruption of *Arabidopsis histone deacetylase 1* (*AtHD1*) expression. Genetics 165, 399–409. 14504245 10.1093/genetics/165.1.399 PMC1462737

[B66] Tirajoh A. Aung T. S. McKay A. B. Plant A. L. (2005). Stress-responsive α*-dioxygenase* expression in tomato roots. J. Exp. Bot. 56, 713–723. 10.1093/jxb/eri038 15618300

[B67] Tombuloglu G. Tombuloglu H. Sakcali M. S. Unver T. (2015). High-throughput transcriptome analysis of barley (*Hordeum vulgare*) exposed to excessive boron. Gene 557, 71–81. 10.1016/j.gene.2014.12.012 25498907

[B68] Turan M. A. Taban N. Taban S. (2009). Effect of calcium on the alleviation of boron toxicity and localization of boron and calcium in cell wall of wheat. Not. Bot. Hort. Agrobot. Cluj. 37, 99–103.

[B69] Turner B. M. (2002). Cellular memory and the histone code. Cell 111, 285–291. 10.1016/S0092-8674(02)01080-2 12419240

[B70] Vantini J. S. Dedemo G. C. Jovino Gimenez D. F. Fonseca L. F. Tezza R. I. Mutton M. A.. (2015). Differential gene expression in drought-tolerant sugarcane roots. Genet. Mol. Res. 14, 7196–7207. 10.4238/2015.June.29.13 26125930

[B71] Wang C. Liu Z. (2006). *Arabidopsis* ribonucleotide reductases are critical for cell cycle progression, DNA damage repair, and plant development. Plant Cell 18, 350–365. 10.1105/tpc.105.037044 16399800 PMC1356544

[B72] Wang H. Y. Lee M. M. Schiefelbein J. W. (2002). Regulation of the cell expansion gene *RHD3* during *Arabidopsis* development. Plant Physiol. 129, 638–649. 10.1104/pp.002675 12068108 PMC161690

[B73] Wang J. Nakazato T. Sakanishi K. Yamada O. Tao H. Saito I. (2006). Single-step microwave digestion with HNO_3_ alone for determination of trace elements in coal by ICP spectrometry. Talanta 68, 1584–1590. 10.1016/j.talanta.2005.08.034 18970502

[B74] Wang Z. Y. Shen K. Zhang F. S. (1995). Effect of boron on nucleic acid metabolism in *Brassica napus* L. Acta Phytophysiol. Sin. 21, 189–194.

[B75] Xu M. Xie W. Huang M. (2012). Overexpression of *PeRHD3* alters the root architecture in *Populus*. Biochem. Biophys. Res. Commun. 424, 239–244. 10.1016/j.bbrc.2012.06.083 22732403

[B76] Xu X. M. Lin H. Maple J. Björkblom B. Alves G. Larsen J. P.. (2010). The *Arabidopsis* DJ-1a protein confers stress protection through cytosolic SOD activation. J. Cell Sci. 123, 1644–1651. 10.1242/jcs.063222 20406884

[B77] Xu X. M. Møller S. G. (2010). ROS removal by DJ-1: *Arabidopsis* as a new model to understand Parkinson's disease. Plant Signal. Behav. 5, 1034–1046. 10.4161/psb.5.8.12298 20671441 PMC3115190

[B78] Zhang Y. Xiao Y. Du F. Cao L. Dong H. Ren H. (2011). *Arabidopsis* VILLIN4 is involved in root hair growth through regulating actin organization in a Ca^2+^-dependent manner. New Phytol. 190, 667–682. 10.1111/j.1469-8137.2010.03632.x 21275995

[B79] Zhou C. P. Qi Y. P. You X. Yang L. T. Guo P. Ye X.. (2013). Leaf cDNA-AFLP analysis of two citrus species differing in manganese tolerance in response to long-term manganese-toxicity. BMC Genomics 14:621. 10.1186/1471-2164-14-621 24034812 PMC3847489

[B80] Zhu Y. Dong A. Shen W. H. (2012). Histone variants and chromatin assembly in plant abiotic stress responses. Biochim. Biophys. Acta 1819, 343–348. 10.1016/j.bbagrm.2011.07.012 24459736

[B81] Zidenga T. Leyva-Guerrero E. Moon H. Siritunga D. Sayre R. (2012). Extending cassava root shelf life *via* reduction of reactive oxygen species production. Plant Physiol. 159, 1396–1407. 10.1104/pp.112.200345 22711743 PMC3425186

