# Supplementary material for: Long-Term Boron-Excess-Induced Alterations of Gene Profiles in Roots of Two Citrus Species Differing in Boron-Tolerance Revealed by cDNA-AFLP
Source: Front Plant Sci. 2016 Jun 24;7:898. doi: 10.3389/fpls.2016.00898 (PMC4919357; doi:10.3389/fpls.2016.00898)
Supplement: Supplementary file 1 [file DataSheet1.DOC]

**Supplementary Material**

**Table S1 | Specific primer pairs used for qRT-PCR expression analysis.**

| **TDF # or gene** | **Forward primers (5´→3´)** | **Reverse primers (5´→3´)** |
| --- | --- | --- |
| 73-2 | AAGACACCGAGCGGTTCACT | TGCCATGCTAAACACCCACC |
| 150-1 | CAGTATCGGCTACGCAAGGA | TTGTCAAGGAAGGTTGTGGG |
| 5-1 | TTGCTGCTCTTCACCTTGGC | GAGTCCTGAGTAACCCTAACATCCT |
| 53-4 | GAGGCAAGGCAATCACTCA | GCCGACTTTCTGGCAAACAACA |
| 112-1 | AGTCCCAAGCCATCTGAATC | CATGCCCTAATGACCGTAGA |
| 26-2 | GCCGATTGTCGTTGTATGG | CTATGTCCGCGTTGTTCCTT |
| 61-1 | TCACGGCTCCGTTGATTAG | GTTATGGCGGTTCCACAATC |
| 65-1 | TCTCACAACGCCTCCAAACG | CGTGCTCTTCCCGCTACTTT |
| 148-1 | TCGGAATCAGCCAGGAAGCA | TCGAGTCAGCCGAGTCGAAGAA |
| 230-1 | GCAACCCGAATGGCTGTGAA | TGAGTAACGGCCAAAGCAC |
| 89-2 | GATGGTCTGGGCACTTTCTT | ATCCAATTCCGGGCTTCCT |
| 150-2 | CCCCAGATAATGAGGCAGACA | CTGAGTAACGACGGAAATGACG |
| 12-1 | TGAGACATTCTCCGACTACCCA | CAGCAAACTGGTGGAAGCAA |
| 137-2 | GATCCAATTCGCCATCACC | ATGAGTCCTGAGTAAGCCAAGC |
| 251-2 | CGCTCGGTGAACATCTTGGT | GCAGCGATAGACTCATCCTTGT |
| *Actin* | AGAACTATGAACTGCCTGATGGC | GCTTGGAGCAAGTGCTGTGATT |
